# Supplementary material for: Multimorbidity and mortality in an older, rural black South African population cohort with high prevalence of HIV findings from the HAALSI Study
Source: BMJ Open. 2021 Sep 15;11(9):e047777. doi: 10.1136/bmjopen-2020-047777 (PMC8444254; doi:10.1136/bmjopen-2020-047777)
Supplement: Supplementary data [file bmjopen-2020-047777supp001.pdf]

Supplementary table 1. Frequency of individual morbidities in individuals with multimorbidity, overall and by sex, age group and HIV status

	Multimorbidity	Sex		Age group (years)					Non-HIV multimorbidity	HIV multimorbidity
		Women	Men	40-49	50-59	60-69	70-79	80+		
	n=3157	n=1770	n=1387	n=507	n=885	n=855	n=555	n=355	n=2242	n=915
Hypertension (%)	2395 (75.9)	1393 (78.7)	1002 (72.2)	292 (57.6)	652 (73.7)	678 (79.3)	467 (84.1)	306 (86.2)	1909 (85.2)	486 (53.1)
Diabetes (%)	534 (16.9)	309 (17.5)	225 (16.2)	46 (9.1)	135 (15.3)	165 (19.3)	123 (22.2)	65 (18.3)	455 (20.3)	79 (8.6)
Dyslipidaemia (%)	1686 (58.1)	919 (56.1)	767 (60.6)	287 (61.6)	480 (59.3)	479 (60.2)	276 (53.1)	164 (52.7)	1280 (62.2)	406 (48.1)
Anaemia (%)	1655 (55.2)	939 (56.2)	716 (53.9)	274 (56.3)	438 (52.1)	445 (54.9)	302 (58.4)	196 (57.3)	1126 (53.3)	529 (59.7)
HIV (%)	915 (29.0)	509 (28.8)	406 (29.3)	256 (50.5)	346 (39.1)	222 (26.0)	79 (14.2)	12 (3.4)		
Angina (%)	428 (13.6)	281 (15.9)	147 (10.6)	47 (9.3)	119 (13.5)	127 (14.9)	74 (13.3)	61 (17.2)	350 (15.6)	78 (8.5)
Chronic bronchitis (%)	27 (0.9)	12 (0.7)	15 (1.1)	5 (1.0)	9 (1.0)	5 (0.6)	4 (0.7)	4 (1.1)	18 (0.8)	9 (1.0)
Depression (%)	752 (24.2)	450 (25.8)	302 (22.1)	78 (15.5)	159 (18.2)	213 (25.2)	156 (28.8)	146 (42.3)	613 (27.8)	139 (15.3)
Post-traumatic stress disorder (%)	222 (7.1)	141 (8.1)	81 (5.9)	31 (6.2)	65 (7.4)	58 (6.9)	39 (7.2)	29 (8.4)	173 (7.9)	49 (5.4)
Alcohol dependence (%)	56 (1.8)	10 (0.6)	46 (3.3)	9 (1.8)	16 (1.8)	24 (2.8)	5 (0.9)	2 (0.6)	42 (1.9)	14 (1.5)

254 individuals had missing data on dyslipidaemia

159 individuals had missing data on anaemia

2 individuals had missing data on chronic bronchitis

49 individuals had missing data on depression

48 individuals had missing data on post-traumatic stress disorder

1 individual had missing data on alcohol dependence

Wade AN, *et al. BMJ Open* 2021; 11:e047777. doi: 10.1136/bmjopen-2020-047777

Supplementary table 2. Odds ratios for death within two years for any multimorbidity vs no multimorbidity

	Total (n=4452)[a]			Women (n=2435)			Men (n=2017)		
	Model 1	Model 2	Model 3	Model 1	Model 2	Model 3	Model 1	Model 2	Model 3
Any multimorbidity	1.77 (1.29-2.44)	1.75 (1.27-2.41)	1.58 (1.14-2.19)	2.40 (1.35-4.25)	2.34 (1.32-4.16)	2.05 (1.14-3.69)	1.51 (1.02-2.23)	1.51 (1.02-2.24)	1.38 (0.92-2.05)
Male sex	1.88 (1.46-2.43)	2.33 (1.74-3.11)	2.33 (1.73-3.14)						
Age	1.05 (1.04-1.06)	1.05 (1.04-1.06)	1.03 (1.02-1.05)	1.06 (1.04-1.07)	1.05 (1.03-1.07)	1.03 (1.01-1.05)	1.05 (1.03-1.06)	1.05 (1.03-1.06)	1.04 (1.02-1.05)
Marital status									
Never married		Reference	Reference		Reference	Reference		Reference	Reference
Previously married		1.06 (0.51-2.18)	1.28 (0.61-2.68)		1.50 (0.35-6.40)	1.62 (0.37-7.07)		0.87 (0.37-2.07)	1.09 (0.45-2.61)
Currently married		0.67 (0.33-1.38)	0.88 (0.42-1.83)		0.67 (0.15-3.01)	0.79 (0.17-3.62)		0.67 (0.29-1.55)	0.88 (0.38-2.05)
Educational attainment									
No formal education		Reference	Reference		Reference	Reference		Reference	Reference
Some primary education		0.92 (0.69-1.24)	1.01 (0.74-1.37)		0.86 (0.53-1.40)	1.01 (0.62-1.66)		0.97 (0.66-1.42)	1.03 (0.70-1.52)
Some secondary education		1.02 (0.62-1.67)	1.13 (0.68-1.88)		1.07 (0.42-2.73)	1.27 (0.49-3.28)		1.03 (0.57-1.86)	1.12 (0.61-2.05)
Secondary or more		0.31 (0.11-0.87)	0.28 (0.10-0.82)		0.26 (0.03-1.96)	0.24 (0.03-1.86)		0.35 (0.10-1.18)	0.33 (0.10-1.13)
Wealth index quintile									
First		Reference	Reference		Reference	Reference		Reference	Reference
Second		1.13 (0.77-1.66)	1.06 (0.72-1.57)		1.17 (0.65-2.13)	1.14 (0.62-2.10)		1.10 (0.67-1.83)	1.03 (0.62-1.72)
Third		1.13 (0.76-1.68)	1.05 (0.70-1.57)		1.11 (0.60-2.05)	0.95 (0.50-1.79)		1.13 (0.68-1.89)	1.10 (0.66-1.86)
Fourth		0.92 (0.61-1.40)	0.94 (0.62-1.44)		0.84 (0.43-1.66)	0.79 (0.40-1.58)		0.98 (0.57-1.66)	1.05 (0.61-1.80)
Fifth		1.18 (0.77-1.80)	1.25 (0.81-1.93)		1.65 (0.86-3.15)	1.68 (0.86-3.26)		0.94 (0.53-1.65)	1.01 (0.57-1.79)
Frailty status									
Non-frail			Reference			Reference			Reference
Pre-frail			1.38 (1.01-1.91)			1.28 (0.76-2.18)			1.49 (1.00-2.24)
Frail			4.72 (3.32-6.71)			5.54 (3.14-9.77)			4.22 (2.67-6.68)

Estimates are given as odds ratios and 95% confidence intervals

Model 1-adjusted for age and sex (in models constructed for total sample)

Model 2-adjusted for age, sex (in models constructed for total sample), marital status, educational attainment and socioeconomic status

Model 3-adjusted for age, sex (in models constructed for total sample), marital status, educational attainment, socioeconomic status and frailty

[a]3 individuals lost to follow up were excluded from the analysis

Supplementary table 3. Odds ratios for death within two years for HIV multimorbidity vs non-HIV multimorbidity

	Total (n=3155)[a]			Women (n=1763)			Men (n=1385)		
	Model 1	Model 2	Model 3	Model 1	Model 2	Model 3	Model 1	Model 2	Model 3
HIV multimorbidity	1.53 (1.09-2.15)	1.41 (0.99-2.00)	1.60 (1.12-2.30)	1.51 (0.87-2.60)	1.36 (0.78-2.39)	1.55 (0.87-2.77)	1.55 (1.00-2.40)	1.48 (0.94-2.34)	1.64 (1.03-2.61)
Male sex	1.71 (1.29-2.27)	1.97 (1.43-2.72)	1.94 (1.39-2.70)						
Age	1.05 (1.04-1.07)	1.05 (1.03-1.06)	1.04 (1.02-1.05)	1.06 (1.04-1.08)	1.05 (1.03-1.08)	1.04 (1.02-1.06)	1.04 (1.03-1.06)	1.04 (1.02-1.06)	1.03 (1.01-1.05)
Marital status									
Never married		Reference	Reference		Reference	Reference		Reference	Reference
Previously married		0.76 (0.36-1.61)	0.91 (0.42-1.96)		1.16 (0.27-5.08)	1.29 (0.29-5.75)		0.62 (0.25-1.53)	0.77 (0.30-1.97)
Currently married		0.57 (0.27-1.19)	0.76 (0.36-1.61)		0.63 (0.14-2.90)	0.79 (0.17-3.74)		0.58 (0.24-1.37)	0.77 (0.32-1.87)
Educational attainment									
No formal education		Reference	Reference		Reference	Reference		Reference	Reference
Some primary education		0.74 (0.53-1.05)	0.83 (0.58-1.18)		0.88 (0.52-1.49)	1.04 (0.61-1.79)		0.67 (0.42-1.05)	0.71 (0.45-1.14)
Some secondary education		1.22 (0.73-2.04)	1.42 (0.84-2.42)		1.32 (0.51-3.42)	1.61 (0.61-4.23)		1.19 (0.64-2.22)	1.35 (0.71-2.57)
Secondary or more		0.35 (0.12-1.00)	0.33 (0.11-0.97)		0.32 (0.04-2.47)	0.32 (0.04-2.52)		0.36 (0.10-1.26)	0.34 (0.10-1.21)
Wealth index quintile									
First		Reference	Reference		Reference	Reference		Reference	Reference
Second		1.14 (0.74-1.75)	1.04 (0.67-1.62)		1.18 (0.63-2.23)	1.16 (0.61-2.22)		1.07 (0.60-1.93)	0.94 (0.51-1.71)
Third		1.11 (0.71-1.73)	0.97 (0.62-1.53)		0.99 (0.51-1.93)	0.84 (0.42-1.67)		1.19 (0.65-2.16)	1.07 (0.58-1.98)
Fourth		0.96 (0.60-1.52)	0.96 (0.60-1.54)		0.81 (0.39-1.67)	0.77 (0.37-1.60)		1.07 (0.58-1.97)	1.12 (0.60-2.09)
Fifth		1.24 (0.77-2.00)	1.31 (0.80-2.13)		1.58 (0.78-3.17)	1.60 (0.78-3.27)		1.02 (0.53-1.97)	1.11 (0.57-2.16)
Frailty status									
Non-frail			Reference			Reference			Reference
Pre-frail			1.41 (0.98-2.02)			1.42 (0.80-2.50)			1.43 (0.89-2.28)
Frail			5.20 (3.50-7.72)			5.42 (2.90-10.16)			5.06 (3.02-8.50)

Estimates are given as odds ratios and 95% confidence intervals

Model 1-adjusted for age and sex (in models constructed for total sample)

Model 2-adjusted for age, sex (in models constructed for total sample), marital status, educational attainment and socioeconomic status

Model 3-adjusted for age, sex (in models constructed for total sample), marital status, educational attainment, socioeconomic status and frailty

[a]2 individuals lost to follow up were excluded from the analysis

Supplementary table 4. Hazard ratios for time to death by multimorbidity status- dyslipidaemia and anaemia excluded from multimorbidity definition

	Any multimorbidity (HR [95% CI])			HIV multimorbidity[a](HR [95% CI])		
	Total[b] (n=4907)	Women (n=2637)	Men (n=2270)	Total[b] (n=1719)	Women (n=1019)	Men (n=700)
Model 1	1.58 (1.32-1.89)	1.50 (1.14-1.97)	1.64 (1.29-2.07)	1.32 (0.73-2.37)	0.89 (0.31-2.52)	1.51 (0.73-3.10)
Model 2	1.57 (1.31-1.88)	1.50 (1.14-1.98)	1.63 (1.29-2.07)	1.23 (0.68-2.22)	0.83 (0.29-2.36)	1.41 (0.68-2.94)
Model 3	1.47 (1.23-1.77)	1.41 (1.07-1.86)	1.53 (1.21-1.95)	1.90 (1.02-3.57)	1.13 (0.36-3.49)	2.13 (1.00-4.57)

Model 1-adjusted for age

Model 2-adjusted for age, marital status, education status and socioeconomic status

Model 3-adjusted for age, marital status, education status, socioeconomic status and frailty

[a] adjusted for HIV multimorbidity-time interaction
[b] adjusted for sex and age-sex interaction term

Wade AN, *et al. BMJ Open* 2021; 11:e047777. doi: 10.1136/bmjopen-2020-047777
