# Supplementary material for: Multimorbidity and mortality in an older, rural black South African population cohort with high prevalence of HIV findings from the HAALSI Study
Source: BMJ Open. 2021 Sep 15;11(9):e047777. doi: 10.1136/bmjopen-2020-047777 (PMC8444254; doi:10.1136/bmjopen-2020-047777)
Supplement: Author's manuscript [file bmjopen-2020-047777.draft_revisions.pdf]

BMJ Open

BMJ Open is committed to open peer review. As part of this commitment we make the peer review history of every article we publish publicly available.

When an article is published we post the peer reviewers' comments and the authors' responses online. We also post the versions of the paper that were used during peer review. These are the versions that the peer review comments apply to.

The versions of the paper that follow are the versions that were submitted during the peer review process. They are not the versions of record or the final published versions. They should not be cited or distributed as the published version of this manuscript.

BMJ Open is an open access journal and the full, final, typeset and author-corrected version of record of the manuscript is available on our site with no access controls, subscription charges or pay-per-view fees (http://bmjopen.bmj.com).

If you have any questions on BMJ Open's open peer review process please email info.bmjopen@bmj.com

BMJ Open

Multimorbidity and mortality in an older, rural Black South African population cohort with high prevalence of HIV- findings from the HAALSI study

Journal:	*BMJ Open*
Manuscript ID	bmjopen-2020-047777
Article Type:	Original research
Date Submitted by the Author:	08-Dec-2020
Complete List of Authors:	Wade, Alisha; University of the Witwatersrand Faculty of Health Sciences, MRC/Wits Rural Public Health and Health Transitions Research Unit, School of Public Health<br>Payne, Collin F.; Australian National University, School of Demography, Research School of Social Sciences<br>Berkman, Lisa; Harvard University T H Chan School of Public Health, Harvard Centre for Population and Development Studies; University of the Witwatersrand Faculty of Health Sciences, MRC/Wits Rural Public Health and Health Transitions Research Unit, School of Public Health<br>Chang, Angela; University of Southern Denmark, Department of Clinical Research; University of Southern Denmark, Danish Institute for Advanced Study<br>Gómez-Olivé, F. Xavier; University of the Witwatersrand Faculty of Health Sciences, MRC/Wits Rural Public Health and Health Transitions Research Unit, School of Public Health<br>Kabudula, Chodziwadziwa; University of the Witwatersrand Faculty of Health Sciences, MRC/Wits Rural Public Health and Health Transitions Research Unit, School of Public Health<br>Kahn, Kathleen; University of the Witwatersrand Faculty of Health Sciences, MRC/Wits Rural Public Health and Health Transitions Research Unit, School of Public Health; Umea University, Umea Centre for Global Health Research<br>Salomon, Joshua ; Stanford University School of Medicine, Centre for Health Policy<br>Tollman, Stephen; University of the Witwatersrand Faculty of Health Sciences, MRC/Wits Rural Public Health and Health Transitions Research Unit, School of Public Health; Umea University, Umea Centre for Global Health Research<br>Witham, Miles; Newcastle University, AGE Research Group, NIHR Newcastle Biomedical Research Centre, Translational Clinical Research Institute; Newcastle Upon Tyne Hospitals NHS Foundation Trust<br>Davies, Justine ; University of Birmingham, Institute of Applied Health Research; Stellenbosch University, Centre for Global Surgery, Department of Global Health
Keywords:	PUBLIC HEALTH, EPIDEMIOLOGY, GERIATRIC MEDICINE

SCHOLARONE™
Manuscripts

I, the Submitting Author has the right to grant and does grant on behalf of all authors of the Work (as defined in the below author licence), an exclusive licence and/or a non-exclusive licence for contributions from authors who are: i) UK Crown employees; ii) where BMJ has agreed a CC-BY licence shall apply, and/or iii) in accordance with the terms applicable for US Federal Government officers or employees acting as part of their official duties; on a worldwide, perpetual, irrevocable, royalty-free basis to BMJ Publishing Group Ltd ("BMJ") its licensees and where the relevant Journal is co-owned by BMJ to the co-owners of the Journal, to publish the Work in this journal and any other BMJ products and to exploit all rights, as set out in our licence.

The Submitting Author accepts and understands that any supply made under these terms is made by BMJ to the Submitting Author unless you are acting as an employee on behalf of your employer or a postgraduate student of an affiliated institution which is paying any applicable article publishing charge ("APC") for Open Access articles. Where the Submitting Author wishes to make the Work available on an Open Access basis (and intends to pay the relevant APC), the terms of reuse of such Open Access shall be governed by a Creative Commons licence – details of these licences and which Creative Commons licence will apply to this Work are set out in our licence referred to above.

Other than as permitted in any relevant BMJ Author's Self Archiving Policies, I confirm this Work has not been accepted for publication elsewhere, is not being considered for publication elsewhere and does not duplicate material already published. I confirm all authors consent to publication of this Work and authorise the granting of this licence.

For peer review only

Multimorbidity and mortality in an older, rural Black South African population cohort with high prevalence of HIV-findings from the HAALSI study

Authors: Alisha N. Wade DPhil[1], Collin F. Payne PhD[2], Lisa F. Berkman PhD[1,3], Angela Y. Chang ScD[4,5], F. Xavier Gómez-Olivé PhD[1], Chodziwadziwa W. Kabudula PhD[1], Kathleen Kahn PhD[1,6,7], Joshua A Salomon PhD[8], Stephen M. Tollman PhD[1,6,7], Miles D. Witham PhD[1,9*] and Justine I. Davies MD[1,10, 11*]

*joint senior authors

Author affiliations: [1]MRC/Wits Rural Public Health and Health Transitions Research Unit, School of Public Health, Faculty of Health Sciences, University of the Witwatersrand, Johannesburg, South Africa; [2]School of Demography, Research School of Social Sciences, Australian National University, Canberra, Australian Capital Territory, Australia; [3]Harvard Centre for Population and Development Studies, Harvard T.H. Chan School of Public Health, Cambridge, Massachusetts, USA; [4]Department of Clinical Research, University of Southern Denmark, Odense, Denmark; [5]Danish Institute for Advanced Study, University of Southern Denmark, Odense, Denmark; [6]Umeå Centre for Global Health Research, Division of Epidemiology and Global Health, Department of Public Health and Clinical Medicine, Umeå University, Umeå, Sweden; [7]INDEPTH Network, Accra, Ghana; [8]Centre for Health Policy, Stanford University, Stanford, California, USA; [9]AGE Research Group, NIHR Newcastle Biomedical Research Centre, Translational Clinical Research Institute, Newcastle University and Newcastle-upon-Tyne NHS Trust, Newcastle upon Tyne, UK; [10]Institute of Applied Health Research,

For peer review only
For peer review only - http://bmjopen.bmj.com/site/about/guidelines.xhtml

University of Birmingham, Birmingham, UK; [11]Centre for Global Surgery, Department of Global Health, Stellenbosch University, Stellenbosch, South Africa.

Corresponding author: Alisha N. Wade MBBS, DPhil, MRC/Wits Rural Public Health and Health Transitions Research Unit, School of Public Health, University of the Witwatersrand, 27 St. Andrew's Road, Parktown, Johannesburg, 2193, South Africa.

Tel:+27 11 717 2511; Fax:+27 86 765 2753; Email: alisha.wade@wits.ac.za

For peer review only

Word count

Abstract: (limit 300 words): 261

Main text: (limit 4000 words): 3439

Tables/Figures (limit 5): 5

For peer review only - http://bmjopen.bmj.com/site/about/guidelines.xhtml

ABSTRACT

Objectives: Multimorbidity is associated with mortality in high-income countries. Our objective was to investigate the relationship between multimorbidity (≥2 chronic medical conditions) and all-cause mortality in an older, rural Black South African population. We further investigated the relationship between HIV multimorbidity (HIV as part of the multimorbidity cluster) and mortality, while testing for the effect of frailty in all models.

Design: Population cohort study.

Setting: Agincourt sub-district of Mpumalanga province, South Africa.

Participants: 4445 individuals (54.7% female), aged ≥40 years and resident in the study area.

Primary and secondary outcome measures: The primary outcome measure was time to death and the secondary outcome measure was likelihood of death within two years of the initial study visit. Mortality was determined during annual population surveillance updates.

Results: 3157 individuals (70.9%) had multimorbidity; 29% of these had HIV. In models adjusted for age and sociodemographic factors, multimorbidity was associated with greater risk of death (women: HR 1.72; 95%CI:1.18-2.50; men: HR 1.46; 95%CI:1.09-1.95) and greater odds of dying within two years (women: OR 2.34; 95%CI:1.32-4.16;

For peer review only

For peer review only - http://bmjopen.bmj.com/site/about/guidelines.xhtml

men: OR 1.51; 95%CI:1.02-2.24). HIV multimorbidity was associated with increased risk of death compared with non-HIV multimorbidity in men (HR 1.93; 95%CI:1.05-3.54), but not in women. Further adjustment for frailty slightly attenuated the associations between multimorbidity and mortality risk (women: HR 1.55; 95%CI:1.06-2.26; men: HR 1.36; 95%CI:1.01-1.82), but slightly increased associations between HIV multimorbidity and mortality risk.

Conclusions: Multimorbidity is associated with mortality in this older Black South African population. Health systems which currently focus on HIV should be reorganised to optimise identification and management of other prevalent chronic diseases.

Keywords: multimorbidity, South Africa, population health, chronic disease, mortality

For peer review only

For peer review only - http://bmjopen.bmj.com/site/about/guidelines.xhtml

STRENGTHS AND LIMITATIONS OF THIS STUDY

- This is, to our knowledge, the first study of multimorbidity and mortality in sub-Saharan Africa and the first to compare mortality between those with HIV multimorbidity and those with non-HIV multimorbidity in any setting.
- Multimorbidity was determined using standardised self-report and objective measures.
- The study was conducted in a large population-based cohort with ascertainment of mortality outcome data in over 99% of participants.
- The clinical severity of morbidities was not assessed and more severe morbidities might be expected to have a greater impact on mortality.
- There was a higher proportion of frailty in the individuals who were excluded due to missing data which may have diluted the impact of frailty on the relationship between multimorbidity and death.

For peer review only

INTRODUCTION

Multimorbidity, commonly defined as the presence of two or more chronic conditions [1,2], has become an increasingly important part of the public health research agenda in sub-Saharan Africa [3-5]. As the population ages, chronic non-communicable diseases such as hypertension, diabetes and ischaemic heart disease have become more common [6]. This trend is influenced by the increase in obesity linked to sedentary lifestyles and consumption of high calorie convenience foods. As in high-income countries, these chronic conditions rarely occur in isolation, with estimates of non-communicable disease multimorbidity varying between 23% and 69% in population-based studies in older adults in sub-Saharan African countries [3,5,7,8]. Multimorbidity in many sub-Saharan African countries is, however, distinguished from that seen in high-income settings by the high prevalence of HIV co-occurring with non-communicable diseases and younger age of onset [9-11], with potential implications for patient outcomes and the way in which multimorbidity is managed by the health care system.

Although evidence from high-income countries suggests that multimorbidity is associated with a higher risk of mortality in older adults [12], it is unclear if this holds true in sub-Saharan Africa where disease patterns and interactions with other ageing syndromes may differ, influenced by the complex health transitions underway in this region [6]. A multimorbidity disease cluster that includes HIV, even if treated, may, for example, confer a higher risk of death than one that does not, given the chronic inflammation and treatment toxicities associated with HIV. Conversely, individuals with

For peer review only - http://bmjopen.bmj.com/site/about/guidelines.xhtml

HIV may have more frequent contact with the health care system and consequently have better control of co-existing non-communicable conditions [13]. Frailty, an ageing syndrome characterised by the progressive loss of ability to withstand physiologic stressors [14], appears to be more prevalent in sub-Saharan Africa than in many high-income countries [15]. Although it is associated with both multimorbidity and mortality in older African populations [16], it is unknown whether frailty affects the relationship between multimorbidity and mortality in these populations or whether it predicts additional mortality risk beyond that provided by multimorbidity alone.

Our objective was to investigate the relationship between multimorbidity and all-cause mortality risk in older Black adults in a rural South African community. We defined older adults as those 40 years and above, given that phenotypic characteristics associated with ageing can occur in sub-Saharan Africa at younger ages than in high-income countries [16,17] and the lower life expectancy in many countries in this region. We hypothesised that multimorbidity was associated with all-cause mortality and performed exploratory subgroup analyses to investigate whether mortality risk differed when HIV was a component of the multimorbidity cluster. We further explored whether frailty modified the ability of multimorbidity to predict death.

METHODS

Study setting and sample

The study used data from Health and Ageing in Africa- a Longitudinal Study in an INDEPTH community (HAALSI) which has previously been described in detail [18]. In

For peer review only - http://bmjopen.bmj.com/site/about/guidelines.xhtml

brief, HAALSI is a longitudinal cohort study which recruited individuals ≥ 40 years and permanently resident in the Agincourt Health and socio-Demographic Surveillance System (HDSS) in the Agincourt sub-district of rural Mpumalanga, northeast South Africa [19]. The Agincourt HDSS is hosted by the MRC/Wits Rural Public Health and Health Transitions Research Unit which annually enumerates its population and documents all births, deaths and migrations, consequently ensuring robust denominators. Of the 12875 eligible individuals, 6281 were randomly selected and 5059 of these individuals enrolled in the study cohort.

Study visits

Baseline study visits took place between November 2014 and November 2015. Trained fieldworkers visited participants in their homes and collected data on sociodemographic variables and self-reported health status and risk factors using Computer-Assisted Personal Interviews (CAPI) and performed clinical assessments including blood pressure, measures of physical performance and point of care biomarkers. Dried blood spots were also collected for assessment of HIV status. The survey instruments were translated from English into Shangaan, the local language, and responses were back-translated into English to ensure reliability.

Definition of variables

Sociodemographic variables

Sociodemographic variables were self-reported and included age (as a continuous variable and calculated as the difference between the date of birth and date of

For peer review only - http://bmjopen.bmj.com/site/about/guidelines.xhtml

interview), marital status (classified as never married, previously married and currently married) and educational attainment (classified as no formal education, at least some primary education, at least some secondary education and completed secondary education or more). Principal components analysis was used to create a wealth index from household characteristics and asset ownership [20].

Multimorbidity

Participants were considered to have multimorbidity if they had two or more [1,2] of the chronic illnesses listed below [3]. We further classified the subset of individuals who had HIV as one of their chronic conditions as having HIV multimorbidity. The chronic illnesses included were selected to ensure comparability with Health and Retirement Survey sister studies as well as to obtain further data on conditions which are prevalent in the study area [18].

Chronic illnesses

The ten included chronic illnesses were defined by a combination of self-report and objective measures [3]. Individuals who reported existing diagnoses of hypertension, diabetes and dyslipidaemia were classified as having these conditions. In addition, the following objective measures were used for classification: hypertension –mean systolic blood pressure ≥140 mmHg or mean diastolic blood pressure ≥90 mmHg calculated from the second and third of three consecutive measurements during the home visit [21]; diabetes mellitus – fasting glucose ≥ 7 mmol/l or random glucose ≥11.1 mmol/l on point of care testing during the home visit (CareSens N monitor; i-SENS, Seoul, South

For peer review only - http://bmjopen.bmj.com/site/about/guidelines.xhtml

Korea) [22] and dyslipidaemia – total cholesterol>6.21 mmol/L, high-density lipoprotein <1.19 mmol/L, low-density lipoprotein >4.1 mmol/L or triglycerides>2.25 mmol/L on point of care testing (CardioChek PA; PTS Diagnostics, Whitestown, IN, USA) [3]. Anaemia was defined as haemoglobin <12 g/dl in women and <13 g/dl in men on point of care testing (Hemocue Hb 201 + analyser; Haemocue, Sweden) [23]. Individuals were considered HIV positive if dried blood spots were positive on screening (Vironostika Uniform 11; Biomeriuex, France) and subsequent confirmatory tests (Roche Elecsys; Roche, USA).

Angina was defined using the Rose criteria [24] and chronic bronchitis was defined as a self-reported daily cough, productive of phlegm, for at least three months per year for at least two successive years [25]. Participants were classified as having depression if they identified three or more symptoms on the Centre for Epidemiological Studies-Depression (CES-D) scale [26], while post-traumatic stress disorder (PTSD) was defined as a score ≥ 4 on the Breslau scale [27]. Individuals were considered to have alcohol dependence if they gave affirmative answers to two or more questions on the CAGE screening questionnaire [28].

Physical function and determination of frailty

We assessed phenotypic frailty status using a locally adapted version of Fried's frailty criteria [29], which has demonstrated construct and predictive validity [16]. Participants scored one point for each of slow walking speed (lowest gait speed quintile [0.46m/s]), low muscle strength (lowest quintile of grip strength [<21.6kg in men and <16.4kg in

For peer review only - http://bmjopen.bmj.com/site/about/guidelines.xhtml

women] assessed using a Baseline® digital Smedley hand dynamometer [Fabrication Enterprises, Hong Kong]), body mass index <18.5 kg/m^2, low activity level (lowest quintile for weekly metabolic equivalents calculated from the Global Physical Activity Questionnaire [30] [<30 metabolic equivalents/week in men and <50 metabolic equivalents/week in women]) and self-reported exhaustion based on a positive response to the question “much of the time you could not get going” from the CES-D scale. Individuals with a total score of ‘0’ were classified as non-frail, those with a total of ‘1’ or ‘2’ as pre-frail and those with a total of ‘3’ or more or those who were physically unable to complete a component of the assessment were classified as frail.

Determination of outcome measures

Our primary outcome was time to death, while we secondarily investigated likelihood of death within two years of the initial study visit. All-cause mortality was determined during the annual HDSS census update rounds [19] and the date of death was ascertained through an interview with a household member of the decedent.

Statistical analysis

Continuous variables were summarised using medians and interquartile ranges and categorical variables as percentages. Mann Whitney and Chi-squared tests were used to compare continuous and categorical variables respectively between groups defined by multimorbidity status.

For peer review only - http://bmjopen.bmj.com/site/about/guidelines.xhtml

We constructed Cox proportional hazards models to investigate the relationship between age-and sex-adjusted multimorbidity status and risk of death in the entire sample and in age-adjusted models stratified by sex. Models were further sequentially adjusted for sociodemographic variables (marital status, educational attainment and wealth quintile) and frailty status, variables which were associated with mortality in previous research in this population [16,31]. Age-sex interaction was statistically significant and was therefore included in models of the entire sample. Individuals were censored at the date of their 2018 census visit or at the date of permanent out-migration from the study area. Any individuals lost to follow up prior to the 2018 census round were censored at the date of their last census visit.

Similar models were constructed to explore the relationship between HIV multimorbidity and risk of death in the subgroup of individuals with multimorbidity. The interaction between HIV multimorbidity and time was statistically significant and was therefore included in the models. Cox models satisfied the assumption of proportional hazards using Schoenfeld residual plots.

Logistic regression models were used to investigate the association between age- and sex-adjusted multimorbidity (both any multimorbidity and HIV multimorbidity) and mortality within 2 years in the entire sample and age-adjusted multimorbidity and 2-year mortality in models stratified by sex. Models were sequentially adjusted as in the Cox proportional hazards models and the areas under the curve (AUCs) were calculated for each model.

For peer review only - http://bmjopen.bmj.com/site/about/guidelines.xhtml

Sensitivity analyses of the primary outcome were conducted in which the chronic conditions with the highest degree of missingness were excluded from the definition of multimorbidity.

P<0.05 was considered statistically significant. Analyses were performed using STATA version 14.2 (StataCorp, USA).

Ethical considerations

Informed consent was obtained from all participants in Shangaan, the local language. Ethical approval was obtained from the Human Research Ethics Committee (Medical) of the University of the Witwatersrand (ref. M141159), Institutional Review Board of the Harvard T.H. Chan School of Public Health, Office of Human Research Administration (ref. C13–1608–02) and Mpumalanga Province Research and Ethics Committee. The research conformed to the principles in the Declaration of Helsinki.

Patient and public involvement

Prior to the initiation of the HAALSI study, an extensive process of community engagement was led by the Agincourt Office of Public Engagement. This included meetings with the Community Advisory Group, nominated by Community Development Forums and civic and traditional leadership structures to discuss planned research activities. Feedback on the results of this study will be included in the annual feedback of study results to villagers and community leaders.

For peer review only

For peer review only - http://bmjopen.bmj.com/site/about/guidelines.xhtml

RESULTS

Six hundred and four (11.9%) cohort members were excluded from this analysis due to missing data, the majority of whom (94.7%) were missing data on multimorbidity status. Missing multimorbidity status was primarily due to missing data on dyslipidaemia and anaemia which resulted from occasional malfunctioning of the measurement device in high ambient temperatures, rather than selection bias. There was no difference in age between those who were included and excluded (p=0.295), while slightly more men were excluded (13.9% vs 10.2%; p<0.001). Frailty distribution differed between the two groups (p<0.001), with a greater proportion of frail individuals among those excluded (39.2% vs 10.3%). Three of the 4445 individuals in the study sample were lost to follow up prior to their two-year study visit; data were available on all other members of the study sample for a minimum of two years after the baseline study visit.

Multimorbidity was present in 3157 individuals (70.9%) in the study sample at the time of the baseline study visit (Table 1). People with multimorbidity were slightly older than those without (median age 61 vs 60 years; p<0.001) and there were more women than men in the multimorbidity group (56.1% vs 51.4%; p=0.004). Multimorbidity was associated with frailty status, with more individuals with multimorbidity in the pre-frail and frail categories (p<0.001). Median follow up time was 3.3 years (interquartile range 3.2-3.5 years), during which time 459 people died.

For peer review only - http://bmjopen.bmj.com/site/about/guidelines.xhtml

Table 1. Sociodemographic, frailty and mortality characteristics overall and by morbidity status

	Total (n=4445)	No multimorbidity (n=1298; 29.1%)	Multimorbidity (n=3157; 70.9%)	p value	Non-HIV multimorbidity (n=2242; 71.0%)	HIV multimorbidity (n=915; 29.0%)	p value
Age (years)	61 (52-71)	60 (50-70)	61 (52-72)	<0.001	64 (55-74)	55 (48-62)	<0.001
Female (%)	2437 (54.7)	667 (51.4)	1770 (56.1)	0.004	1261 (56.2)	509 (55.6)	0.752
Marital status (%)							
Never married	226 (5.1)	80 (6.2)	146 (4.6)		86 (3.8)	60 (6.6)	
Previously married	1934 (43.4)	467 (36.0)	1467 (46.5)		967 (43.1)	500 (54.6)	
Currently married	2295 (51.5)	751 (57.9)	1544 (48.9)	<0.001	1189 (53.0)	355 (38.8)	<0.001
Educational attainment (%)							
No formal education	2036 (45.7)	562 (43.3)	1474 (46.7)		1092 (48.7)	382 (41.8)	
Some primary education	1550 (34.8)	444 (34.2)	1106 (35.0)		784 (35.0)	322 (35.2)	
Some secondary education	505 (11.3)	163 (12.6)	342 (10.8)		201 (9.0)	141 (15.4)	
Secondary or more	364 (8.2)	129 (9.9)	235 (7.4)	0.008	165 (7.4)	70 (7.7)	<0.001
Wealth index quintile(%)							
1st	915 (20.5)	273 (21.0)	642 (20.3)		417 (18.6)	225 (24.6)	
2nd	887 (199)	257 (19.8)	630 (20.0		438 (19.5)	192 (21.0)	
3rd	878 (19.7)	264 (20.3)	614 (19.5)		429 (19.1)	185 (20.2)	
4th	881 (19.8)	246 (19.0)	635 (20.1)		465 (20.7)	170 (18.6)	
5th	894 (20.1)	258 (19.9)	636 (20.2)	0.868	493 (22.0)	143 (15.6)	<0.001
Frailty status (%)							
Non-frail	2347 (52.7)	811 (62.5)	1536 (48.7)		1003 (44.7)	533 (58.3)	
Pre-frail	1648 (37.0)	398 (30.7)	1250 (39.6)		925 (41.3)	325 (35.5)	
Frail	460 (10.3)	89 (6.9)	371 (11.8)	<0.001	314 (14.0)	57 (6.2)	<0.001

For peer review only

Nine hundred and fifteen (29%) of the 3157 individuals with multimorbidity had HIV as one of their multimorbid conditions. Those with HIV multimorbidity were younger (median age 55 vs 64 years; $p<0.001$) and less frail than those with non-HIV multimorbidity ($p<0.001$).

After adjustment for age and sociodemographic factors, multimorbidity, compared with no multimorbidity, was associated with greater risk of death in both women (HR 1.72; 95%CI: 1.18-2.50) and men (HR 1.46; 95%CI: 1.09-1.95) (Table 2). The results remained significant after further adjustment for frailty status (women HR 1.55; 95%CI: 1.06-2.26 and men HR 1.36; 95% CI: 1.01-1.82). HIV multimorbidity, compared to non-HIV multimorbidity and similarly adjusted for age and sociodemographic factors, was only associated with an increased risk of death in men (HR 1.93; 95%CI: 1.05-3.54). Nevertheless, HIV multimorbidity was associated with a significantly increased risk of death for both men and women after further adjustment for frailty status.

For peer review only - http://bmjopen.bmj.com/site/about/guidelines.xhtml

Table 2. Hazard ratios for risk of death by multimorbidity status

	Any multimorbidity (HR [95% CI])			HIV multimorbidity[a] (HR [95% CI])		
	Total[b] (n=4455)	Women (n=2437)	Men (n=2018)	Total[b] (n=3157)	Women (n=1770)	Men (n=1387)
Model 1	1.56 (1.24-1.97)	1.72 (1.18-2.49)	1.47 (1.10-1.97)	2.08 (1.30-3.35)	1.97 (0.91-4.26)	2.10 (1.15-3.84)
Model 2	1.55 (1.23-1.95)	1.72 (1.18-2.50)	1.46 (1.09-1.95)	1.92 (1.19-3.10)	1.85 (0.85-4.04)	1.93 (1.05-3.54)
Model 3	1.42 (1.13-1.79)	1.55 (1.06-2.26)	1.36 (1.01-1.82)	2.59 (1.57-4.27)	2.75 (1.19-6.37)	2.34 (1.26-4.34)

Model 1-adjusted for age
Model 2-adjusted for age, marital status, education status and socioeconomic status
Model 3-adjusted for age, marital status, education status, socioeconomic status and frailty

[a] adjusted for HIV multimorbidity-time interaction

[b] adjusted for sex and age-sex interaction term

For peer review only

In a model fully adjusted for age, socio-demographic factors and frailty, there was a 58% increase in the odds of death within two years in individuals with multimorbidity compared to those without multimorbidity (OR 1.58; 95%CI:1.14-2.19) (Table 3). There was a 76.7% chance (95%CI 73.9-79.5%) that this model would discriminate between those who died within two years and those who did not, with the addition of frailty resulting in a small but significant improvement in discriminant ability over the model in which multimorbidity was adjusted only for age and sociodemographic factors (Fig. 1). In fully adjusted models stratified by sex, women with multimorbidity were twice as likely to be dead within two years of the baseline visit as those without multimorbidity (OR 2.05; 95%CI 1.14-3.69); there was no significant association in men (OR 1.38; 0.92-2.05). There was a 79.2% chance in women (95% CI 74.9-83.6%) and a 72.5% chance in men (95%CI 68.6-76.4%) that these adjusted models would discriminate between those who died within two years and those who did not. The addition of frailty resulted in a marginal improvement, in women, in discriminant ability over the model in which multimorbidity was adjusted only for age and sociodemographic factors.

For peer review only

Table 3. Odds ratios for death within two years for any multimorbidity vs no multimorbidity

	Total (n=4452)[a]			Women (n=2435)			Men (n=2017)		
	Model 1	Model 2	Model 3	Model 1	Model 2	Model 3	Model 1	Model 2	Model 3
Any multimorbidity	1.77 (1.29-2.44)	1.75 (1.27-2.41)	1.58 (1.14-2.19)	2.40 (1.35-4.25)	2.34 (1.32-4.16)	2.05 (1.14-3.69)	1.51 (1.02-2.23)	1.51 (1.02-2.24)	1.38 (0.92-2.05)
Male sex	1.88 (1.46-2.43)	2.33 (1.74-3.11)	2.33 (1.73-3.14)						
Age	1.05 (1.04-1.06)	1.05 (1.04-1.06)	1.03 (1.02-1.05)	1.06 (1.04-1.07)	1.05 (1.03-1.07)	1.03 (1.01-1.05)	1.05 (1.03-1.06)	1.05 (1.03-1.06)	1.04 (1.02-1.05)
Marital status									
Never married		Reference	Reference		Reference	Reference		Reference	Reference
Previously married		1.06 (0.51-2.18)	1.28 (0.61-2.68)		1.50 (0.35-6.40)	1.62 (0.37-7.07)		0.87 (0.37-2.07)	1.09 (0.45-2.61)
Currently married		0.67 (0.33-1.38)	0.88 (0.42-1.83)		0.67 (0.15-3.01)	0.79 (0.17-3.62)		0.67 (0.29-1.55)	0.88 (0.38-2.05)
Educational attainment									
No formal education		Reference	Reference		Reference	Reference		Reference	Reference
Some primary education		0.92 (0.69-1.24)	1.01 (0.74-1.37)		0.86 (0.53-1.40)	1.01 (0.62-1.66)		0.97 (0.66-1.42)	1.03 (0.70-1.52)
Some secondary education		1.02 (0.62-1.67)	1.13 (0.68-1.88)		1.07 (0.42-2.73)	1.27 (0.49-3.28)		1.03 (0.57-1.86)	1.12 (0.61-2.05)
Secondary or more		0.31 (0.11-0.87)	0.28 (0.10-0.82)		0.26 (0.03-1.96)	0.24 (0.03-1.86)		0.35 (0.10-1.18)	0.33 (0.10-1.13)
Wealth quintile									
First		Reference	Reference		Reference	Reference		Reference	Reference
Second		1.13 (0.77-1.66)	1.06 (0.72-1.57)		1.17 (0.65-2.13)	1.14 (0.62-2.10)		1.10 (0.67-1.83)	1.03 (0.62-1.72)
Third		1.13 (0.76-1.68)	1.05 (0.70-1.57)		1.11 (0.60-2.05)	0.95 (0.50-1.79)		1.13 (0.68-1.89)	1.10 (0.66-1.86)
Fourth		0.92 (0.61-1.40)	0.94 (0.62-1.44)		0.84 (0.43-1.66)	0.79 (0.40-1.58)		0.98 (0.57-1.66)	1.05 (0.61-1.80)
Fifth		1.18 (0.77-1.80)	1.25 (0.81-1.93)		1.65 (0.86-3.15)	1.68 (0.86-3.26)		0.94 (0.53-1.65)	1.01 (0.57-1.79)
Frailty status									
Non-frail			Reference			Reference			Reference
Pre-frail			1.38 (1.01-1.91)			1.28 (0.76-2.18)			1.49 (1.00-2.24)
Frail			4.72 (3.32-6.71)			5.54 (3.14-9.77)			4.22 (2.67-6.68)

Estimates are given as odds ratios and 95% confidence intervals
Model 1-adjusted for age and sex (in models constructed for total sample)
Model 2-adjusted for age, sex (in models constructed for total sample), marital status, educational attainment and socioeconomic status
Model 3-adjusted for age, sex (in models constructed for total sample), marital status, educational attainment, socioeconomic status and frailty
[a]3 individuals lost to follow up were excluded from the analysis

For peer review only - http://bmjopen.bmj.com/site/about/guidelines.xhtml

In similarly fully adjusted models, there was a 60% increase in the odds of death within two years in individuals with HIV multimorbidity compared to those with non-HIV multimorbidity (OR 1.60; 95% CI 1.12-2.30) (Table 4). Adjustment of HIV multimorbidity for frailty, in addition to age and sociodemographic factors, significantly improved the discriminant ability of the model (Fig 1). In sex-stratified models, there was a 64% increase in the odds of death within two years in men with HIV multimorbidity compared to those with non-HIV multimorbidity (OR 1.64; 95%CI 1.03-2.61), while there was no association in women. As with any multimorbidity, models that included frailty marginally improved, in women, upon the discriminant ability of models in which HIV multimorbidity was adjusted only for age and sociodemographic factors.

For peer review only - http://bmjopen.bmj.com/site/about/guidelines.xhtml

Table 4. Odds ratios for death within two years for HIV multimorbidity vs non-HIV multimorbidity

	Total (n=3155)[a]			Women (n=1763)			Men (n=1385)		
	Model 1	Model 2	Model 3	Model 1	Model 2	Model 3	Model 1	Model 2	Model 3
HIV multimorbidity	1.53 (1.09-2.15)	1.41 (0.99-2.00)	1.60 (1.12-2.30)	1.51 (0.87-2.60)	1.36 (0.78-2.39)	1.55 (0.87-2.77)	1.55 (1.00-2.40)	1.48 (0.94-2.34)	1.64 (1.03-2.61)
Male sex	1.71 (1.29-2.27)	1.97 (1.43-2.72)	1.94 (1.39-2.70)						
Age	1.05 (1.04-1.07)	1.05 (1.03-1.06)	1.04 (1.02-1.05)	1.06 (1.04-1.08)	1.05 (1.03-1.08)	1.04 (1.02-1.06)	1.04 (1.03-1.06)	1.04 (1.02-1.06)	1.03 (1.01-1.05)
Marital status		Reference							
Never married		0.76 (0.36-1.61)	Reference		Reference	Reference		Reference	Reference
Previously married		0.57 (0.27-1.19)	0.91 (0.42-1.96)		1.16 (0.27-5.08)	1.29 (0.29-5.75)		0.62 (0.25-1.53)	0.77 (0.30-1.97)
Currently married			0.76 (0.36-1.61)		0.63 (0.14-2.90)	0.79 (0.17-3.74)		0.58 (0.24-1.37)	0.77 (0.32-1.87)
Educational attainment									
No formal education		Reference	Reference		Reference	Reference		Reference	Reference
Some primary education		0.74 (0.53-1.05)	0.83 (0.58-1.18)		0.88 (0.52-1.49)	1.04 (0.61-1.79)		0.67 (0.42-1.05)	0.71 (0.45-1.14)
Some secondary education		1.22 (0.73-2.04)	1.42 (0.84-2.42)		1.32 (0.51-3.42)	1.61 (0.61-4.23)		1.19 (0.64-2.22)	1.35 (0.71-2.57)
Secondary or more		0.35 (0.12-1.00)	0.33 (0.11-0.97)		0.32 (0.04-2.47)	0.32 (0.04-2.52)		0.36 (0.10-1.26)	0.34 (0.10-1.21)
Wealth quintile									
First		Reference	Reference		Reference	Reference		Reference	Reference
Second		1.14 (0.74-1.75)	1.04 (0.67-1.62)		1.18 (0.63-2.23)	1.16 (0.61-2.22)		1.07 (0.60-1.93)	0.94 (0.51-1.71)
Third		1.11 (0.71-1.73)	0.97 (0.62-1.53)		0.99 (0.51-1.93)	0.84 (0.42-1.67)		1.19 (0.65-2.16)	1.07 (0.58-1.98)
Fourth		0.96 (0.60-1.52)	0.96 (0.60-1.54)		0.81 (0.39-1.67)	0.77 (0.37-1.60)		1.07 (0.58-1.97)	1.12 (0.60-2.09)
Fifth		1.24 (0.77-2.00)	1.31 (0.80-2.13)		1.58 (0.78-3.17)	1.60 (0.78-3.27)		1.02 (0.53-1.97)	1.11 (0.57-2.16)
Frailty status									
Non-frail			Reference			Reference			Reference
Pre-frail			1.41 (0.98-2.02)			1.42 (0.80-2.50)			1.43 (0.89-2.28)
Frail			5.20 (3.50-7.72)			5.42 (2.90-10.16)			5.06 (3.02-8.50)

Estimates are given as odds ratios and 95% confidence intervals

Model 1-adjusted for age and sex (in models constructed for total sample)

Model 2-adjusted for age, sex (in models constructed for total sample), marital status, educational attainment and socioeconomic status

Model 3-adjusted for age, sex (in models constructed for total sample), marital status, educational attainment, socioeconomic status and frailty

[a]2 individuals lost to follow up were excluded from the analysis

For peer review only

For peer review only - http://bmjopen.bmj.com/site/about/guidelines.xhtml

In sensitivity analyses, dyslipidaemia and anaemia were excluded from the chronic conditions that contributed to the definition of multimorbidity. Fewer cohort members were excluded and there was a final sample size of 4907 individuals, of whom 1719 (35%) had multimorbidity; 637 (37.1%) of these had HIV multimorbidity. Multimorbidity remained significantly associated with a higher risk of death in both women (HR 1.41; 95%CI 1.07-1.86) and men (HR 1.53; 95%CI 1.21-1.95) after adjustment for age, sociodemographic factors and frailty. Adjusted HIV multimorbidity, when compared to non-HIV multimorbidity, was associated with a marginally higher risk of death in men only (HR 2.13; 95%CI 1.00-4.57) (Supplementary Table 1).

DISCUSSION

In this older, rural, Black South African cohort, multimorbidity was associated with greater risk of death in both women and men. Men who had HIV as part of their multimorbidity cluster had a higher risk of death than those with non-HIV multimorbidity. The relationship between multimorbidity and death remained robust after adjusting for a range of sociodemographic variables as well as after adjusting for frailty status, itself a strong independent risk factor for mortality. Addition of frailty to models of multimorbidity adjusted for age and other sociodemographic factors slightly increased the ability of these models to discriminate between those who died within two years and those who did not.

Although there is a paucity of literature on multimorbidity and mortality in sub-Saharan Africa, our results are in keeping with similar population-based studies in high-income

For peer review only - http://bmjopen.bmj.com/site/about/guidelines.xhtml

and other middle-income countries, with several studies reporting associations between multimorbidity and mortality. One-year mortality rate ratios for individuals ≥ 40 years in a Canadian population ranged between 2 and 6 [32], although in this study, multimorbidity was defined more conservatively as three or more chronic conditions, rather than two. In a Chinese population aged 45 years and older, multimorbidity was associated with a 95% increase in risk of death during a four year follow up [33], while in Brazilian adults ≥60 years, risk for all-cause mortality over 16 years in individuals with two or more chronic diseases ranged between 36% and 105% in women and 58-59% in men [34]. In contrast, an insurance claims-based study in Germans aged 65 years and older identified only a small association between multimorbidity and mortality during a 5 year follow up period [35]. This study, however, defined multimorbidity as 2 or more of 46 conditions, some of which were not chronic and would also be expected to have a relatively small effect on mortality, which was reflected in the range of hazard ratios of the individual conditions in the study (0.70-2.29 in women and 0.66-1.95 in men).

This is, to our knowledge, the first study of the relationship between multimorbidity and mortality in sub-Saharan Africa, a region experiencing rapid health transition and with an ageing population and increasing prevalence of multimorbidity. It is also, again to our knowledge, the first study to compare mortality in those with HIV as part of their multimorbidity cluster to those with non-HIV multimorbidity. The study was conducted in a large population-based cohort, using standardised self-reported and objectively assessed morbidities. Our follow up was also robust, with ascertainment of mortality outcome data in over 99% of participants. Our study was, however, limited by

For peer review only - http://bmjopen.bmj.com/site/about/guidelines.xhtml

For peer review only

considering only whether a morbidity was present and not its clinical severity and more severe morbidities might be expected to have a greater impact on mortality. We also considered multimorbidity as a homogeneous concept, and there is some evidence that multimorbidity clusters, defined by groupings of similar diseases [36], are associated with differential mortality risk [37].There was a higher proportion of frailty in the individuals who were excluded due to missing data which may have diluted the impact of frailty on the relationship between multimorbidity and death.

Our study has implications for both clinical care and health policy in South Africa and other sub-Saharan African countries undergoing health transitions with similar demographics and disease patterns. Clinicians should be aware that the presence of multimorbidity may increase the risk of death in older adults in the short term, even in the absence of impairments in physical function. Aggressive screening and risk factor modification should therefore be employed in individuals with single morbidities who are at high risk of developing a second condition. The sex differential in the relationship between HIV multimorbidity and mortality may reflect the higher mortality seen in men with HIV [38], which may itself be related to women accessing HIV care and adhering to treatment more frequently than men [39] and highlighting the need to specifically target men in testing and treatment initiation and adherence campaigns. Our finding that frailty status only modestly enhances the ability of multimorbidity, adjusted for age and sociodemographic factors, to discriminate between those who did and did not die within two years suggests that frailty assessments may be most useful in assessing mortality risk in older adults without multimorbidity.

For peer review only - http://bmjopen.bmj.com/site/about/guidelines.xhtml

Our results also have implications for the way in which health systems are structured in South Africa. HIV care frequently serves as the entry point into chronic health care for older adults and adherence to HIV treatment programmes may be associated with improvements in non-communicable disease outcomes [40]. Our findings suggest, however, a significant need to broaden the scope of chronic care beyond HIV and ensure that health services are equipped to detect and manage a wide range of chronic conditions in community-dwelling older adults.

CONCLUSION

Our study indicates greater risk of all-cause mortality in older adults with multimorbidity in a sub-Saharan African country. Further research is, however, required to investigate the relationship in this population between mortality and multimorbidity disease clusters as well as the clinical severity of multimorbid conditions. In the interim, models of care which optimise identification and management of multimorbidity, regardless of HIV status, need to be developed [41].

For peer review only

ACKNOWLEDGEMENTS

We would like to acknowledge the residents of the Agincourt sub-district and Health and socio-Demographic Surveillance System who participated in this study.

MDW acknowledges support from the NIHR Newcastle Biomedical Research Centre.

AUTHOR CONTRIBUTIONS

ANW, LB, FXGO, CWK, KK, JAS and SMT were involved in design and data collection for HAALSI. ST, KK, CWK and FXGO established the HDSS and mortality data collection system. ANW, MDW and JID designed this study and ANW performed the statistical analysis. ANW wrote the first draft of the manuscript. All authors critically revised the manuscript and approved the final version.

COMPETING INTERESTS STATEMENT

The authors have no competing interests to report.

FUNDING

This work was supported by the National Institute on Ageing at the National Institutes of Health (grant number 1P01AG041710-01A1); the Department of Science and Innovation, South Africa, the University of the Witwatersrand, and the Medical Research Council, South Africa, and previously the Wellcome Trust, UK (grant numbers 058893/Z/99/A; 069683/Z/02/Z; 085477/Z/08/Z; 085477/B/08/Z) to the MRC/Wits Rural Public Health and Health Transitions Research Unit and Agincourt Health and socio-

For peer review only - http://bmjopen.bmj.com/site/about/guidelines.xhtml

Demographic Surveillance System, a node of the South African Population Research Infrastructure Network (SAPRIN); the Fogarty International Centre of the National Institutes of Health (grant number K43TW010698) to ANW; the Australian Research Council Discovery Early Career Award and the Australian National University Futures Scheme to CFP.

This paper describes the views of the authors and does not necessarily represent the official views of the National Institutes of Health (USA).

The funders had no role in study design, data collection, analysis and interpretation, report writing or the decision to submit this article for publication.

DATA SHARING STATEMENT

The HAALSI baseline data are publicly available at the Harvard Centre for Population and Development Studies (HCPDS) programme website [www.haalsi.org]. Data are also accessible through the Inter-university Consortium for Political and Social Research (ICPSR) at the University of Michigan [www.icpsr.umich.edu] and the INDEPTH Data Repository [http://www.indepth-ishare.org/index.php/catalog/113]. Mortality data are available upon request.

For peer review only - http://bmjopen.bmj.com/site/about/guidelines.xhtml

REFERENCES

1. Barnett K, Mercer SW, Norbury M, et al. Epidemiology of multimorbidity and implications for health care, research, and medical education: a cross-sectional study. *Lancet.* 2012;380:37-43. doi: 10.1016/S0140-6736(12)60240-2.
2. Multimorbidity: a priority for global health research. London: Academy of Medical Sciences;2018.
3. Chang AY, Gómez-Olivé FX, Payne C, et al. Chronic multimorbidity among older adults in rural South Africa. *BMJ Glob Health.* 2019;4:e001386. doi: 10.1136/bmjgh-2018-001386.
4. Nimako BA, Baiden F, Sackey SO, et al. Multimorbidity of chronic diseases among adult patients presenting to an inner-city clinic in Ghana. *Global Health.* 2013;9:61. doi: 10.1186/1744-8603-9-61.
5. Hien H, Berthe A, Drabo MK, et al. Prevalence and patterns of multimorbidity among the elderly in Burkina Faso: cross-sectional study. *Trop Med Int Health.* 2014;19:1328-1333. doi: 10.1111/tmi.12377.
6. Gouda HN, Charlson F, Sorsdahl K, et al. Burden of non-communicable diseases in sub-Saharan Africa, 1990-2017: results from the Global Burden of Disease Study 2017. *Lancet Glob Health.* 2019;7:e1375-e1387. doi: 10.1016/S2214-109X(19)30374-2.
7. Garin N, Koyanagi A, Chatterji S, et al. Global multimorbidity patterns: a cross-sectional, population-based, multi-country study. *J Gerontol A Biol Sci Med Sci.* 2016;71:205-214. doi: 10.1093/gerona/glv128.

For peer review only - http://bmjopen.bmj.com/site/about/guidelines.xhtml

8. Odland ML, Payne C, Witham MD, et al. Epidemiology of multimorbidity in conditions of extreme poverty: a population-based study of older adults in rural Burkina Faso. *BMJ Glob Health.* 2020;5:e002096. doi: 10.1136/bmjgh-2019-002096.

9. Magodoro IM, Esterhuizen TM, Chivese T. A cross-sectional, facility based study of comorbid non-communicable diseases among adults living with HIV infection in Zimbabwe. *BMC Res Notes.* 2016;9:379. doi: 10.1186/s13104-016-2187-z.

10. Kansiime S, Mwesigire D, Mugerwa H. Prevalence of non-communicable diseases among HIV positive patients on antiretroviral therapy at Joint Clinical Research Centre, Lubowa, Uganda. *PLoS One.* 2019;14:e0221022. doi: 10.1371/journal.pone.0221022.

11. Oni T, Youngblood E, Boulle A, et al. Patterns of HIV, TB, and non-communicable disease multi-morbidity in peri-urban South Africa- a cross sectional study. *BMC Infect Dis.* 2015;15:20. doi: 10.1186/s12879-015-0750-1.

12. Nunes BP, Flores TR, Mielke GI, et al. Multimorbidity and mortality in older adults: a systematic review and meta-analysis. *Arch Gerontol Geriatr.* 2016;67:130-138. doi: 10.1016/j.archger.2016.07.008.

13. Manne-Goehler J, Montana L, Gomez-Olive FX, et al. The ART advantage: health care utilization for diabetes and hypertension in rural South Africa. *J Acquir Immune Defic Syndr.* 2017;75:561-567. doi: 10.1097/QAI.0000000000001445.

14. Clegg A, Young J, Iliffe S, et al. Frailty in elderly people. *Lancet.* 2013;381:752-762. doi: 10.1016/S0140-6736(12)62167-9.

For peer review only - http://bmjopen.bmj.com/site/about/guidelines.xhtml

15. Majid Z, Welch C, Davies J, et al. Global frailty: the role of ethnicity, migration and socioeconomic factors. *Maturitas.* 2020;139:33-41. doi: 10.1016/j.maturitas.2020.05.010.

16. Payne CF, Wade A, Kabudula CW, et al. Prevalence and correlates of frailty in an older rural African population: findings from the HAALSI cohort study. *BMC Geriatr.* 2017;17:293. doi: 10.1186/s12877-017-0694-y.

17. Payne CF, Gomez-Olive FX, Kahn K, et al. Physical function in an ageing population in rural South Africa: findings from HAALSI and cross-national comparisons with HRS sister studies. *J Gerontol B Psychol Sci Soc Sci.* 2017;72:665-679. doi: 10.1093/geronb/gbx030.

18. Gomez-Olive FX, Montana L, Wagner RG, et al. Cohort profile: Health and Ageing in Africa: a Longitudinal Study of an INDEPTH Community in South Africa (HAALSI). Int J Epidemiol. 2018;47:689-690j. doi: 10.1093/ije/dyx247.

19. Kahn K, Collinson MA, Gomez-Olive FX, et al. Profile: Agincourt health and socio-demographic surveillance system. *Int J Epidemiol.* 2012;41:988-1001. doi: 10.1093/ije/dys115.

20. Vyas S, Kumaranayake L. Constructing socio-economic status indices: how to use principal components analysis. *Health Policy Plan.* 2006;21:459-468. doi: 10.1093/heapol/czl029.

21. Chobanian AV, Bakris GL, Black HR, et al. Seventh report of the Joint National Committee on prevention, detection, evaluation, and treatment of high blood pressure. *Hypertension.* 2003;42:1206-1252. doi: 10.1161/01.HYP.0000107251.49515.c2.

For peer review only - http://bmjopen.bmj.com/site/about/guidelines.xhtml

22. American Diabetes Association. Classification and diagnosis of diabetes: standards of medical care in diabetes-2020. *Diabetes Care.* 2020;43(Suppl 1):S14-S31. doi: 10.2337/dc20-S002.

23. Shisana O, Labadarios D, Rehle T, et al. South African National Health and Nutrition Examination Survey (SANHANES-1). Cape Town: HSRC Press; 2013.

24. Rose G, McCartney P, Reid DD. Self-administration of a questionnaire on chest pain and intermittent claudication. *Br J Prev Soc Med.* 1977;31:42-48. doi: 10.1136/jech.31.1.42.

25. Ehrlich RI, White N, Norman R, et al. Predictors of chronic bronchitis in South African adults. *Int J Tuberc Lung Dis.* 2004;8:369-376.

26. Radloff LS. The CES-D scale:a self-report depression scale for research in the general population. *Appl Psychol Meas.* 1977;1:385-401. doi: 10.1177/014662167700100306.

27. Breslau N, Peterson EL, Kessler RC, et al. Short screening scale for DSM-IV posttraumatic stress disorder. *Am J Psychiatry.* 1999;156:908-911. doi: 10.1176/ajp.156.6.908.

28. Ewing JA. Detecting alcoholism. The CAGE questionnaire. *JAMA.* 1984;252:1905-1907. doi: 10.1001/jama.252.14.1905.

29. Fried LP, Tangen CM, Walston J, et al. Frailty in older adults: evidence for a phenotype. *J Gerontol A Biol Sci Med Sci.* 2001;56:M146-156.

30. World Health Organisation. Global Physical Activity Questionnaire analysis guide. https://www.who.int/ncds/surveillance/steps/GPAQ/en/. Accessed October 30, 2020.

For peer review only

31. Gómez-Olivé FX, Thorogood M, Bocquier P, et al. Social conditions and disability related to the mortality of older people in rural South Africa. *Int J Epidemiol.* 2014;43:1531-1541. doi: 10.1093/ije/dyu093.

32. Ryan BL, Allen B, Zwarenstein M, et al. Multimorbidity and mortality in Ontario, Canada: a population-based retrospective cohort study. *J Comorb.* 2020;10:2235042X20950598. doi: 10.1177/2235042X20950598.

33. Zhang Y, Zhou L, Liu S, et al. Prevalence, correlates and outcomes of multimorbidity among the middle-aged and elderly: findings from the China Health and Retirement Longitudinal Study. *Arch Gerontol Geriatr.* 2020;90:104135. doi: 10.1016/j.archger.2020.104135.

34. Roman Lay AA, Ferreira do Nascimento C, Caba Burgos F, et al. Gender differences between multimorbidity and all-cause mortality among older adults. *Curr Gerontol Geriatr Res.* 2020;2020:7816785. doi: 10.1155/2020/7816785.

35. Schafer I, Kaduszkiewicz H, Nguyen TS, et al. Multimorbidity patterns and 5-year overall mortality: results from a claims data-based observational study. *J Comorb.* 2018;8:2235042X18816588. doi: 10.1177/2235042X18816588.

36. Chang AY, Gomez-Olive FX, Manne-Goehler J, et al. Multimorbidity and care for hypertension, diabetes and HIV among older adults in rural South Africa. *Bull World Health Organ.* 2019;97:10-23. doi: 10.2471/BLT.18.217000.

37. Vetrano DL, Roso-Llorach A, Fernandez S, et al. Twelve-year clinical trajectories of multimorbidity in a population of older adults. *Nat Commun.* 2020;11:3223. doi: 10.1038/s41467-020-16780-x.

For peer review only - http://bmjopen.bmj.com/site/about/guidelines.xhtml

38. Madut DB, Park LP, Yao J, et al. Predictors of mortality in treatment experienced HIV-infected patients in northern Tanzania. *PLoS One.* 2020;15:e0240293. doi: 10.1371/journal.pone.0240293.

39. Osler M, Cornell M, Ford N, et al. Population-wide differentials in HIV service access and outcomes in the Western Cape for men as compared to women, South Africa: 2008 to 2018: a cohort analysis. *J Int AIDS Soc.* 2020;23 Suppl 2:e25530. doi: 10.1002/jia2.25530.

40. Manne-Goehler J, Siedner MJ, Montana L, et al. Hypertension and diabetes control along the HIV care cascade in rural South Africa. *J Int AIDS Soc.* 2019;22:e25213. doi: 10.1002/jia2.25213.

41. Ameh S, Gomez-Olive FX, Kahn K, et al. Multilevel predictors of controlled CD4 count and blood pressure in an integrated chronic disease management model in rural South Africa: a panel study. *BMJ Open.* 2020;10:e037580. doi: 10.1136/bmjopen-2020-037580.

For peer review only

For peer review only - http://bmjopen.bmj.com/site/about/guidelines.xhtml

Figure 1. Receiver operating curves for multimorbidity and death within two years

Model 1- any multimorbidity/HIV multimorbidity and age; model 2-any multimorbidity/HIV multimorbidity, marital status, educational attainment and socioeconomic status; model 3-any multimorbidity/HIV multimorbidity, marital status, educational attainment, socioeconomic status and frailty status. Models constructed for total sample were additionally adjusted for sex. AUC: area under the curve

For peer review only

For peer review only - http://bmjopen.bmj.com/site/about/guidelines.xhtml

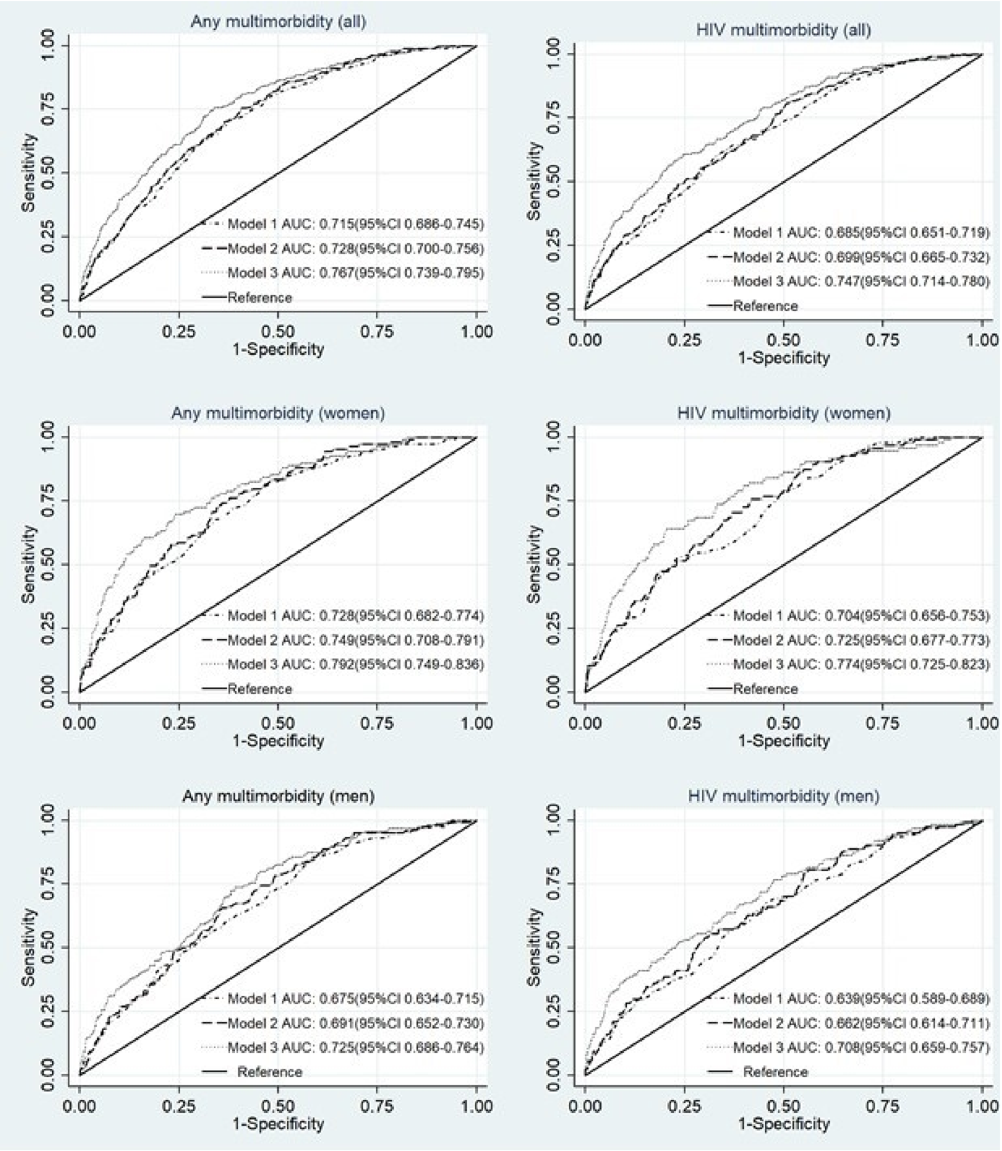

Figure 1. Receiver operating curves for multimorbidity and death within two years
Model 1- any multimorbidity/HIV multimorbidity and age; model 2-any multimorbidity/HIV multimorbidity, marital status, educational attainment and socioeconomic status; model 3-any multimorbidity/HIV multimorbidity, marital status, educational attainment, socioeconomic status and frailty status. Models constructed for total sample were additionally adjusted for sex. AUC: area under the curve

163x189mm (96 x 96 DPI)

Supplementary Table 1. Hazard ratios for time to death by multimorbidity status- dyslipidaemia and anaemia excluded from multimorbidity definition

	Any multimorbidity (HR [95% CI])			HIV multimorbidity[a](HR [95% CI])		
	Total[b] (n=4907)	Women (n=2637)	Men (n=2270)	Total[b] (n=1719)	Women (n=1019)	Men (n=700)
Model 1	1.58 (1.32-1.89)	1.50 (1.14-1.97)	1.64 (1.29-2.07)	1.32 (0.73-2.37)	0.89 (0.31-2.52)	1.51 (0.73-3.10)
Model 2	1.57 (1.31-1.88)	1.50 (1.14-1.98)	1.63 (1.29-2.07)	1.23 (0.68-2.22)	0.83 (0.29-2.36)	1.41 (0.68-2.94)
Model 3	1.47 (1.23-1.77)	1.41 (1.07-1.86)	1.53 (1.21-1.95)	1.90 (1.02-3.57)	1.13 (0.36-3.49)	2.13 (1.00-4.57)

Model 1-adjusted for age

Model 2-adjusted for age, marital status, education status and socioeconomic status

Model 3-adjusted for age, marital status, education status, socioeconomic status and frailty

[a] adjusted for HIV multimorbidity-time interaction

[b] adjusted for sex and age-sex interaction term

For peer review only

STROBE 2007 (v4) Statement—Checklist of items that should be included in reports of *cohort studies*

Section/Topic	Item #	Recommendation	Reported on page #
**Title and abstract**	1	(*a*) Indicate the study's design with a commonly used term in the title or the abstract	1
		(*b*) Provide in the abstract an informative and balanced summary of what was done and what was found	3-4
**Introduction**			
Background/rationale	2	Explain the scientific background and rationale for the investigation being reported	6-7
Objectives	3	State specific objectives, including any prespecified hypotheses	7
**Methods**			
Study design	4	Present key elements of study design early in the paper	7-8
Setting	5	Describe the setting, locations, and relevant dates, including periods of recruitment, exposure, follow-up, and data collection	7-11
Participants	6	(*a*) Give the eligibility criteria, and the sources and methods of selection of participants. Describe methods of follow-up	7-8
		(*b*) For matched studies, give matching criteria and number of exposed and unexposed	n/a
Variables	7	Clearly define all outcomes, exposures, predictors, potential confounders, and effect modifiers. Give diagnostic criteria, if applicable	8-11
Data sources/ measurement	8*	For each variable of interest, give sources of data and details of methods of assessment (measurement). Describe comparability of assessment methods if there is more than one group	8-11
Bias	9	Describe any efforts to address potential sources of bias	13
Study size	10	Explain how the study size was arrived at	8,14
Quantitative variables	11	Explain how quantitative variables were handled in the analyses. If applicable, describe which groupings were chosen and why	11-12
Statistical methods	12	(*a*) Describe all statistical methods, including those used to control for confounding	11-13
		(*b*) Describe any methods used to examine subgroups and interactions	11-12
		(*c*) Explain how missing data were addressed	14
		(*d*) If applicable, explain how loss to follow-up was addressed	14
		(*e*) Describe any sensitivity analyses	13
**Results**			

For peer review only

Participants	13*	(a) Report numbers of individuals at each stage of study—eg numbers potentially eligible, examined for eligibility, confirmed eligible, included in the study, completing follow-up, and analysed	14
		(b) Give reasons for non-participation at each stage	14
		(c) Consider use of a flow diagram	
Descriptive data	14*	(a) Give characteristics of study participants (eg demographic, clinical, social) and information on exposures and potential confounders	14
		(b) Indicate number of participants with missing data for each variable of interest	14
		(c) Summarise follow-up time (eg, average and total amount)	14
Outcome data	15*	Report numbers of outcome events or summary measures over time	14
Main results	16	(*a*) Give unadjusted estimates and, if applicable, confounder-adjusted estimates and their precision (eg, 95% confidence interval). Make clear which confounders were adjusted for and why they were included	16-21
		(*b*) Report category boundaries when continuous variables were categorized	n/a
		(*c*) If relevant, consider translating estimates of relative risk into absolute risk for a meaningful time period	n/a
Other analyses	17	Report other analyses done—eg analyses of subgroups and interactions, and sensitivity analyses	22
**Discussion**			
Key results	18	Summarise key results with reference to study objectives	22
**Limitations**			
Interpretation	20	Give a cautious overall interpretation of results considering objectives, limitations, multiplicity of analyses, results from similar studies, and other relevant evidence	22-25
Generalisability	21	Discuss the generalisability (external validity) of the study results	24
**Other information**			
Funding	22	Give the source of funding and the role of the funders for the present study and, if applicable, for the original study on which the present article is based	26-27

*Give information separately for cases and controls in case-control studies and, if applicable, for exposed and unexposed groups in cohort and cross-sectional studies.

Note: An Explanation and Elaboration article discusses each checklist item and gives methodological background and published examples of transparent reporting. The STROBE checklist is best used in conjunction with this article (freely available on the Web sites of PLoS Medicine at http://www.plosmedicine.org/, Annals of Internal Medicine at http://www.annals.org/, and Epidemiology at http://www.epidem.com/). Information on the STROBE Initiative is available at www.strobe-statement.org.

For peer review only

BMJ Open

Multimorbidity and mortality in an older, rural Black South African population cohort with high prevalence of HIV- findings from the HAALSI study

Journal:	*BMJ Open*
Manuscript ID	bmjopen-2020-047777.R1
Article Type:	Original research
Date Submitted by the Author:	20-Jul-2021
Complete List of Authors:	Wade, Alisha N.; University of the Witwatersrand Faculty of Health Sciences, MRC/Wits Rural Public Health and Health Transitions Research Unit, School of Public Health<br>Payne, Collin F.; Australian National University, School of Demography, Research School of Social Sciences<br>Berkman, Lisa; Harvard University T H Chan School of Public Health, Harvard Centre for Population and Development Studies; University of the Witwatersrand Faculty of Health Sciences, MRC/Wits Rural Public Health and Health Transitions Research Unit, School of Public Health<br>Chang, Angela; University of Southern Denmark, Department of Clinical Research; University of Southern Denmark, Danish Institute for Advanced Study<br>Gómez-Olivé, F. Xavier; University of the Witwatersrand Faculty of Health Sciences, MRC/Wits Rural Public Health and Health Transitions Research Unit, School of Public Health<br>Kabudula, Chodziwadziwa; University of the Witwatersrand Faculty of Health Sciences, MRC/Wits Rural Public Health and Health Transitions Research Unit, School of Public Health<br>Kahn, Kathleen; University of the Witwatersrand Faculty of Health Sciences, MRC/Wits Rural Public Health and Health Transitions Research Unit, School of Public Health; Umea University, Umea Centre for Global Health Research<br>Salomon, Joshua ; Stanford University School of Medicine, Centre for Health Policy<br>Tollman, Stephen; University of the Witwatersrand Faculty of Health Sciences, MRC/Wits Rural Public Health and Health Transitions Research Unit, School of Public Health; Umea University, Umea Centre for Global Health Research<br>Witham, Miles; Newcastle University, AGE Research Group, NIHR Newcastle Biomedical Research Centre, Translational Clinical Research Institute; Newcastle Upon Tyne Hospitals NHS Foundation Trust<br>Davies, Justine ; University of Birmingham, Institute of Applied Health Research; Stellenbosch University, Centre for Global Surgery, Department of Global Health
**Primary Subject Heading**:	Public health
Secondary Subject Heading:	Epidemiology

Keywords:	PUBLIC HEALTH, EPIDEMIOLOGY, GERIATRIC MEDICINE

SCHOLARONE™
Manuscripts

I, the Submitting Author has the right to grant and does grant on behalf of all authors of the Work (as defined in the below author licence), an exclusive licence and/or a non-exclusive licence for contributions from authors who are: i) UK Crown employees; ii) where BMJ has agreed a CC-BY licence shall apply, and/or iii) in accordance with the terms applicable for US Federal Government officers or employees acting as part of their official duties; on a worldwide, perpetual, irrevocable, royalty-free basis to BMJ Publishing Group Ltd ("BMJ") its licensees and where the relevant Journal is co-owned by BMJ to the co-owners of the Journal, to publish the Work in this journal and any other BMJ products and to exploit all rights, as set out in our licence.

The Submitting Author accepts and understands that any supply made under these terms is made by BMJ to the Submitting Author unless you are acting as an employee on behalf of your employer or a postgraduate student of an affiliated institution which is paying any applicable article publishing charge ("APC") for Open Access articles. Where the Submitting Author wishes to make the Work available on an Open Access basis (and intends to pay the relevant APC), the terms of reuse of such Open Access shall be governed by a Creative Commons licence – details of these licences and which Creative Commons licence will apply to this Work are set out in our licence referred to above.

Other than as permitted in any relevant BMJ Author's Self Archiving Policies, I confirm this Work has not been accepted for publication elsewhere, is not being considered for publication elsewhere and does not duplicate material already published. I confirm all authors consent to publication of this Work and authorise the granting of this licence.

For peer review only

Multimorbidity and mortality in an older, rural Black South African population cohort with high prevalence of HIV-findings from the HAALSI study

Authors: Alisha N. Wade DPhil[1], Collin F. Payne PhD[2], Lisa F. Berkman PhD[1,3], Angela Y. Chang ScD[4,5], F. Xavier Gómez-Olivé PhD[1], Chodziwadziwa W. Kabudula PhD[1], Kathleen Kahn PhD[1,6,7], Joshua A Salomon PhD[8], Stephen M. Tollman PhD[1,6,7], Miles D. Witham PhD[1,9*] and Justine I. Davies MD[1,10, 11*]

*joint senior authors

Author affiliations: [1]MRC/Wits Rural Public Health and Health Transitions Research Unit, School of Public Health, Faculty of Health Sciences, University of the Witwatersrand, Johannesburg, South Africa; [2]School of Demography, Research School of Social Sciences, Australian National University, Canberra, Australian Capital Territory, Australia; [3]Harvard Centre for Population and Development Studies, Harvard T.H. Chan School of Public Health, Cambridge, Massachusetts, USA; [4]Department of Clinical Research, University of Southern Denmark, Odense, Denmark; [5]Danish Institute for Advanced Study, University of Southern Denmark, Odense, Denmark; [6]Umeå Centre for Global Health Research, Division of Epidemiology and Global Health, Department of Public Health and Clinical Medicine, Umeå University, Umeå, Sweden; [7]INDEPTH Network, Accra, Ghana; [8]Centre for Health Policy, Stanford University, Stanford, California, USA; [9]AGE Research Group, NIHR Newcastle Biomedical Research Centre, Translational Clinical Research Institute, Newcastle University and Newcastle-upon-Tyne NHS Trust, Newcastle upon Tyne, UK; [10]Institute of Applied Health Research,

For peer review only - http://bmjopen.bmj.com/site/about/guidelines.xhtml

For peer review only

University of Birmingham, Birmingham, UK; [11]Centre for Global Surgery, Department of Global Health, Stellenbosch University, Stellenbosch, South Africa.

Corresponding author: Justine I. Davies MD, Institute of Applied Health Research, Murray Learning Centre, University of Birmingham, Edgbaston, Birmingham, B15 2TT UK. Tel:+44 121 414 3762; Email: j.davies.6@bham.ac.uk

For peer review only

Word count

Abstract: 299 words

Main text: 4101 words

Tables/Figures (limit 5): 4 (main); 4 (supplementary)

For peer review only - http://bmjopen.bmj.com/site/about/guidelines.xhtml

ABSTRACT

Objectives: Multimorbidity is associated with mortality in high-income countries. Our objective was to investigate the relationship between multimorbidity (≥2 of the following chronic medical conditions: hypertension, diabetes, dyslipidaemia, anaemia, HIV, angina, depression, post-traumatic stress disorder, alcohol dependence) and all-cause mortality in an older, rural Black South African population. We further investigated the relationship between HIV multimorbidity (HIV as part of the multimorbidity cluster) and mortality, while testing for the effect of frailty in all models.

Design: Population cohort study.

Setting: Agincourt sub-district of Mpumalanga province, South Africa.

Participants: 4455 individuals (54.7% female), aged ≥40 years (median age 61 years, interquartile range 52-71) and resident in the study area.

Primary and secondary outcome measures: The primary outcome measure was time to death and the secondary outcome measure was likelihood of death within two years of the initial study visit. Mortality was determined during annual population surveillance updates.

Results: 3157 individuals (70.9%) had multimorbidity; 29% of these had HIV. In models adjusted for age and sociodemographic factors, multimorbidity was associated with

For peer review only - http://bmjopen.bmj.com/site/about/guidelines.xhtml

greater risk of death (women: HR 1.72; 95%CI:1.18-2.50; men: HR 1.46; 95%CI:1.09-1.95) and greater odds of dying within two years (women: OR 2.34; 95%CI:1.32-4.16; men: OR 1.51; 95%CI:1.02-2.24). HIV multimorbidity was associated with increased risk of death compared with non-HIV multimorbidity in men (HR 1.93; 95%CI:1.05-3.54), but was not statistically significant in women (HR 1.85; 95%CI:0.85-4.04); when detectable, HIV viral loads were higher in men (p=0.021). Further adjustment for frailty slightly attenuated the associations between multimorbidity and mortality risk (women: HR 1.55; 95%CI:1.06-2.26; men: HR 1.36; 95%CI:1.01-1.82), but slightly increased associations between HIV multimorbidity and mortality risk.

Conclusions: Multimorbidity is associated with mortality in this older Black South African population. Health systems which currently focus on HIV should be reorganised to optimise identification and management of other prevalent chronic diseases.

Keywords: multimorbidity, South Africa, population health, chronic disease, mortality

For peer review only

STRENGTHS AND LIMITATIONS OF THIS STUDY

- This is, to our knowledge, the first study of multimorbidity and mortality in sub-Saharan Africa and the first to compare mortality between those with HIV multimorbidity and those with non-HIV multimorbidity in any setting.
- Multimorbidity was determined using standardised self-report and objective measures.
- The study was conducted in a large population-based cohort with ascertainment of mortality outcome data in over 99% of participants.
- The clinical severity of morbidities was not assessed and more severe morbidities might be expected to have a greater impact on mortality.
- There was a higher proportion of frailty in the individuals who were excluded due to missing data which may have diluted the impact of frailty on the relationship between multimorbidity and death.

For peer review only

For peer review only - http://bmjopen.bmj.com/site/about/guidelines.xhtml

INTRODUCTION

Multimorbidity, commonly defined as the presence of two or more chronic conditions [1,2], has become an increasingly important part of the public health research agenda in sub-Saharan Africa [3-5]. As the population ages, chronic non-communicable diseases such as hypertension, diabetes and ischaemic heart disease have become more common [6]. This trend is influenced by the increase in obesity linked to sedentary lifestyles and consumption of high calorie convenience foods. As in high-income countries, these chronic conditions rarely occur in isolation, with estimates of non-communicable disease multimorbidity varying between 23% and 69% in population-based studies in older adults in sub-Saharan African countries [3,5,7,8]. Multimorbidity in many sub-Saharan African countries is, however, distinguished from that seen in high-income settings by the high prevalence of HIV co-occurring with non-communicable diseases and younger age of onset [9-11], with potential implications for patient outcomes and the way in which multimorbidity is managed by the health care system.

Although evidence from high-income countries suggests that multimorbidity is associated with a higher risk of mortality in older adults [12], it is unclear if this holds true in sub-Saharan Africa where disease patterns and interactions with other ageing syndromes may differ, influenced by the complex health transitions underway in this region [6]. A multimorbidity disease cluster that includes HIV, even if treated, may, for example, confer a higher risk of death than one that does not, given the chronic inflammation and treatment toxicities associated with HIV. Conversely, individuals with

For peer review only - http://bmjopen.bmj.com/site/about/guidelines.xhtml

HIV may have more frequent contact with the health care system and consequently have better control of co-existing non-communicable conditions [13]. Frailty, an ageing syndrome characterised by the progressive loss of ability to withstand physiologic stressors [14], appears to be more prevalent in sub-Saharan Africa than in many high-income countries [15]. Although it is associated with both multimorbidity and mortality in older African populations [16], it is unknown whether frailty affects the relationship between multimorbidity and mortality in these populations or whether it predicts additional mortality risk beyond that predicted by multimorbidity alone.

Our objective was to investigate the relationship between multimorbidity and all-cause mortality risk in older Black adults in a rural South African community. We defined older adults as those 40 years and above, given that phenotypic characteristics associated with ageing can occur in sub-Saharan Africa at younger ages than in high-income countries [16,17] and the lower life expectancy in many countries in this region. We hypothesised that multimorbidity was associated with all-cause mortality and performed exploratory subgroup analyses to investigate whether mortality risk differed when HIV was a component of the multimorbidity cluster. We further explored whether frailty modified the ability of multimorbidity to predict death.

METHODS

Study setting and sample

The study used data from Health and Ageing in Africa- a Longitudinal Study in an INDEPTH community (HAALSI) which has previously been described in detail [18]. In

For peer review only - http://bmjopen.bmj.com/site/about/guidelines.xhtml

brief, HAALSI is a longitudinal cohort study which recruited individuals ≥ 40 years and permanently resident in the Agincourt Health and socio-Demographic Surveillance System (HDSS) in the Agincourt sub-district of rural Mpumalanga, northeast South Africa [19]. The Agincourt HDSS is hosted by the MRC/Wits Rural Public Health and Health Transitions Research Unit which annually enumerates its population and documents all births, deaths and migrations, consequently ensuring robust denominators. Of the 12875 eligible individuals, 6281 were randomly selected and 5059 of these individuals enrolled in the study cohort.

Study visits

Baseline study visits took place between November 2014 and November 2015. Trained fieldworkers visited participants in their homes and collected data on sociodemographic variables and self-reported health status and risk factors using Computer-Assisted Personal Interviews (CAPI) and performed clinical assessments including blood pressure, measures of physical performance and point of care biomarkers. Dried blood spots were also collected for assessment of HIV serostatus and viral load. The survey instruments were translated from English into Shangaan, the local language, and responses were back-translated into English to ensure reliability. Translation was performed by experienced members of the Unit staff with further minor modifications by the fieldworkers who conducted the interviews to ensure the language used was in keeping with the vernacular.

Definition of variables

For peer review only - http://bmjopen.bmj.com/site/about/guidelines.xhtml

Sociodemographic variables

Sociodemographic variables were self-reported and included age (as a continuous variable and calculated as the difference between the date of birth and date of interview), marital status (classified as never married, previously married and currently married) and educational attainment (classified as no formal education, at least some primary education, at least some secondary education and completed secondary education or more). Principal component analysis was used to create a wealth index from household characteristics and asset ownership [20].

Multimorbidity

Participants were considered to have multimorbidity if they had two or more [1,2] of the chronic illnesses listed below [3]. We further classified the subset of individuals who had HIV as one of their chronic conditions as having HIV multimorbidity. The chronic illnesses included were selected to ensure comparability with Health and Retirement Survey sister studies as well as to obtain further data on conditions which are prevalent in the study area [18].

Chronic illnesses

The ten included chronic illnesses were defined by a combination of self-report and objective measures [3]. Individuals who reported existing diagnoses of hypertension, diabetes and dyslipidaemia were classified as having these conditions. In addition, the following objective measures were used for classification: hypertension –mean systolic blood pressure ≥140 mmHg or mean diastolic blood pressure ≥90 mmHg calculated

For peer review only - http://bmjopen.bmj.com/site/about/guidelines.xhtml

from the second and third of three consecutive measurements during the home visit [21]; diabetes mellitus – fasting glucose ≥ 7 mmol/l or random glucose ≥11.1 mmol/l on point of care testing during the home visit (CareSens N monitor; i-SENS, Seoul, South Korea) [22] and dyslipidaemia – total cholesterol>6.21 mmol/L, high-density lipoprotein <1.19 mmol/L, low-density lipoprotein >4.1 mmol/L or triglycerides>2.25 mmol/L on point of care testing (CardioChek PA; PTS Diagnostics, Whitestown, IN, USA) [3]. Anaemia was defined as haemoglobin <12 g/dl in women and <13 g/dl in men on point of care testing (Hemocue Hb 201 + analyser; Haemocue, Sweden) [23]. Individuals were considered HIV positive if dried blood spots were positive on screening (Vironostika Uniform 11; Biomeriuex, France) and subsequent confirmatory tests (Roche Elecsys; Roche, USA). Dried blood spots from individuals who tested positive for HIV were then tested for HIV-1 RNA (BioMérieux NucliSens; lower limit of detection 100 copies/ml).

Angina was defined using the Rose criteria [24] and chronic bronchitis was defined as a self-reported daily cough, productive of phlegm, for at least three months per year for at least two successive years [25]. Participants were classified as having depression if they identified three or more symptoms on the Centre for Epidemiological Studies-Depression (CES-D) scale [26], while post-traumatic stress disorder (PTSD) was defined as a score ≥ 4 on the Breslau scale [27]. Individuals were considered to have alcohol dependence if they gave affirmative answers to two or more questions on the CAGE screening questionnaire [28].

For peer review only - http://bmjopen.bmj.com/site/about/guidelines.xhtml

Physical function and determination of frailty

We assessed phenotypic frailty status using a locally adapted version of Fried's frailty criteria [29], which has demonstrated construct and predictive validity [16]. Participants scored one point for each of slow walking speed (lowest gait speed quintile [0.46m/s]), low muscle strength (lowest quintile of grip strength [<21.6kg in men and <16.4kg in women] assessed using a Baseline® digital Smedley hand dynamometer [Fabrication Enterprises, Hong Kong]), body mass index <18.5 kg/m^2, low activity level (lowest quintile for weekly metabolic equivalents calculated from the Global Physical Activity Questionnaire [30] [<30 metabolic equivalents/week in men and <50 metabolic equivalents/week in women]) and self-reported exhaustion based on a positive response to the question "much of the time you could not get going" from the CES-D scale. Individuals with a total score of '0' were classified as non-frail, those with a total of '1' or '2' as pre-frail and those with a total of '3' or more or those who were physically unable to complete a component of the assessment were classified as frail.

Determination of outcome measures

Our primary outcome was time to death, while we secondarily investigated likelihood of death within two years of the initial study visit. All-cause mortality was determined during the annual HDSS census update rounds [19] and the date of death was ascertained through an interview with a household member of the decedent.

Statistical analysis

For peer review only - http://bmjopen.bmj.com/site/about/guidelines.xhtml

Continuous variables were not normally distributed and were therefore summarised using medians and interquartile ranges (IQRs) while categorical variables were summarised using percentages. Mann Whitney and Chi-squared tests were used to compare continuous and categorical variables respectively between groups defined by inclusion/exclusion in the analysis, sex and multimorbidity status.

We constructed Cox proportional hazards models to investigate the relationship between age-and sex-adjusted multimorbidity status and risk of death in the entire sample and in age-adjusted models stratified by sex. Models were further sequentially adjusted for sociodemographic variables (marital status, educational attainment and wealth quintile) and frailty status, variables which were associated with mortality in previous research in this population [16,31]. Age-sex interaction was statistically significant and was therefore included in models of the entire sample. Individuals were censored at the date of their 2018 census visit or at the date of permanent out-migration from the study area. Any individuals lost to follow up prior to the 2018 census round were censored at the date of their last census visit.

Similar models were constructed to explore the relationship between HIV multimorbidity and risk of death in the subgroup of individuals with multimorbidity. The interaction between HIV multimorbidity and time was statistically significant and was therefore included in the models. Cox models satisfied the assumption of proportional hazards using Schoenfeld residual plots.

For peer review only - http://bmjopen.bmj.com/site/about/guidelines.xhtml

In supplementary analyses, logistic regression models were used to investigate the association between age- and sex-adjusted multimorbidity (both any multimorbidity and HIV multimorbidity) and mortality within 2 years in the entire sample and age-adjusted multimorbidity and 2-year mortality in models stratified by sex. Models were sequentially adjusted as in the Cox proportional hazards models and the areas under the curve (AUCs) were calculated for each model.

Sensitivity analyses of the primary outcome were conducted in which the chronic conditions with the highest degree of missingness were excluded from the definition of multimorbidity.

P<0.05 was considered statistically significant. Analyses were performed using STATA version 14.2 (StataCorp, USA).

Ethical approval

Informed consent was obtained from all participants in Shangaan, the local language. Ethical approval was obtained from the Human Research Ethics Committee (Medical) of the University of the Witwatersrand (ref. M141159), Institutional Review Board of the Harvard T.H. Chan School of Public Health, Office of Human Research Administration (ref. C13–1608–02) and Mpumalanga Province Research and Ethics Committee. The research conformed to the principles in the Declaration of Helsinki.

Patient and public involvement

For peer review only - http://bmjopen.bmj.com/site/about/guidelines.xhtml

Prior to the initiation of the HAALSI study, an extensive process of community engagement was led by the Agincourt Office of Public Engagement. This included meetings with the Community Advisory Group, nominated by Community Development Forums and civic and traditional leadership structures to discuss planned research activities. Feedback on the results of this study will be included in the annual feedback of study results to villagers and community leaders.

RESULTS

Six hundred and four (11.9%) cohort members were excluded from this analysis due to missing data, the majority of whom (94.7%) were missing data on multimorbidity status. Missing multimorbidity status was primarily due to missing data on dyslipidaemia and anaemia which resulted from occasional malfunctioning of the measurement device in high ambient temperatures, rather than selection bias. There was no difference in age between those who were included and excluded (p=0.295), while slightly more men were excluded (13.9% vs 10.2%; p<0.001). Frailty distribution differed between the two groups (p<0.001), with a greater proportion of frail individuals among those excluded (39.2% vs 10.3%). Three of the 4455 individuals in the study sample were lost to follow up prior to their two-year study visit; data were available on all other members of the study sample for a minimum of two years after the baseline study visit.

Multimorbidity was present in 3157 individuals (70.9%) in the study sample at the time of the baseline study visit (Table 1). People with multimorbidity were slightly older than those without (median age 61 vs 60 years; p<0.001) and there were more women than

For peer review only

men in the multimorbidity group (56.1% vs 51.4%; p=0.004). Multimorbidity was associated with frailty status, with more individuals with multimorbidity in the pre-frail and frail categories (p<0.001). Nine hundred and fifteen (29%) of the 3157 individuals with multimorbidity had HIV as one of their multimorbid conditions. Those with HIV multimorbidity were younger (median age 55 vs 64 years; p<0.001) and less frail than those with non-HIV multimorbidity (p<0.001). Just over half of those with HIV multimorbidity had an undetectable viral load. There was no difference in the proportion of women and men with HIV multimorbidity who had an undetectable viral load (52.9% vs 50.7%; p=0.811), but men with HIV multimorbidity and a detectable viral load had a higher median viral load than women in the same category (3700 copies/ml [IQR 790-24000] vs 1600 copies/ml [IQR 480-16000]; p=0.021). While hypertension was the most prevalent individual disease in those with multimorbidity (75.9%), the frequency of individual conditions varied by sex, age group and HIV multimorbidity status; hypertension was the most frequent condition in those with non-HIV multimorbidity, for example, but the second most frequent in those with HIV multimorbidity. (Supplementary table 1).

Median follow up time was 3.3 years (IQR 3.2-3.5 years), during which time 459 people died. The median age of those who died was 71 years (IQR 60-81) and 200 (43.6%) of these were women. Eighty percent of those who died had multimorbidity and of those, 88 (24.0%) had HIV multimorbidity. Approximately 39% of those with HIV multimorbidity who died had an undetectable viral load (Table 2).

For peer review only - http://bmjopen.bmj.com/site/about/guidelines.xhtml

Table 1. Sociodemographic, frailty and mortality characteristics overall and by morbidity status

	Total (n=4455)	No multimorbidity (n=1298; 29.1%)	Multimorbidity (n=3157; 70.9%)	p value	Non-HIV multimorbidity (n=2242; 71.0%)	HIV multimorbidity (n=915; 29.0%)	p value
Age (years)	61 (52-71)	60 (50-70)	61 (52-72)	<0.001	64 (55-74)	55 (48-62)	<0.001
Female (%)	2437 (54.7)	667 (51.4)	1770 (56.1)	0.004	1261 (56.2)	509 (55.6)	0.752
Marital status (%)							
Never married	226 (5.1)	80 (6.2)	146 (4.6)		86 (3.8)	60 (6.6)	
Previously married	1934 (43.4)	467 (36.0)	1467 (46.5)		967 (43.1)	500 (54.6)	
Currently married	2295 (51.5)	751 (57.9)	1544 (48.9)	<0.001	1189 (53.0)	355 (38.8)	<0.001
Educational attainment (%)							
No formal education	2036 (45.7)	562 (43.3)	1474 (46.7)		1092 (48.7)	382 (41.8)	
Some primary education	1550 (34.8)	444 (34.2)	1106 (35.0)		784 (35.0)	322 (35.2)	
Some secondary education	505 (11.3)	163 (12.6)	342 (10.8)		201 (9.0)	141 (15.4)	
Secondary or more	364 (8.2)	129 (9.9)	235 (7.4)	0.008	165 (7.4)	70 (7.7)	<0.001
Wealth index quintile(%)							
1st	915 (20.5)	273 (21.0)	642 (20.3)		417 (18.6)	225 (24.6)	
2nd	887 (199)	257 (19.8)	630 (20.0		438 (19.5)	192 (21.0)	
3rd	878 (19.7)	264 (20.3)	614 (19.5)		429 (19.1)	185 (20.2)	
4th	881 (19.8)	246 (19.0)	635 (20.1)		465 (20.7)	170 (18.6)	
5th	894 (20.1)	258 (19.9)	636 (20.2)	0.868	493 (22.0)	143 (15.6)	<0.001
Frailty status (%)							
Non-frail	2347 (52.7)	811 (62.5)	1536 (48.7)		1003 (44.7)	533 (58.3)	
Pre-frail	1648 (37.0)	398 (30.7)	1250 (39.6)		925 (41.3)	325 (35.5)	
Frail	460 (10.3)	89 (6.9)	371 (11.8)	<0.001	314 (14.0)	57 (6.2)	<0.001
Undetectable viral load[a](%)						475 (51.9)	
Viral load (copies/ml)						2200 (630-18000)	

[a]Two individuals with HIV had insufficient sample for viral load testing. Undetectable viral load was defined as <100 copies/ml. Continuous variables are summarised as medians and interquartile ranges and compared using the Mann-Whitney test. Categorical variables were compared using the Chi-squared test .

For peer review only - http://bmjopen.bmj.com/site/about/guidelines.xhtml

Table 1. Characteristics of participants who died during the follow-up period

	n=459
Age (years)	71 (60-81)
Female (%)	200 (43.6)
Marital status (%)	
Never married	22 (4.8)
Previously married	246 (53.6)
Currently married	191 (41.6)
Educational attainment (%)	
No formal education	278 (60.6)
Some primary education	142 (30.9)
Some secondary education	31 (6.8)
Secondary or more	8 (1.7)
Wealth index quintile (%)	
1st	102 (22.2)
2nd	93 (20.3)
3rd	100 (21.8)
4th	85 (18.5)
5th	79 (17.2)
Frailty status (%)	
Non-frail	141 (30.7)
Pre-frail	187 (40.7)
Frail	131 (28.5)
Multimorbidity (%)	367 (80.0)
HIV multimorbidity (%)	88 (24.0)
Undetectable viral load (%)	34 (38.6)
Viral load (copies/ml)	18000 (1700-37000)

Undetectable viral load was defined as <100 copies/ml. Continuous variables are summarised as medians and interquartile ranges; HIV multimorbidity is presented as a sub-group of those with multimorbidity and data on viral load are for the sub-group with HIV multimorbidity.

For peer review only

After adjustment for age and sociodemographic factors, multimorbidity, compared with no multimorbidity, was associated with greater risk of death in both women (HR 1.72; 95%CI: 1.18-2.50) and men (HR 1.46; 95%CI: 1.09-1.95) (Table 3). The results remained significant after further adjustment for frailty status (women HR 1.55; 95%CI: 1.06-2.26 and men HR 1.36; 95% CI: 1.01-1.82). HIV multimorbidity, compared to non-HIV multimorbidity and similarly adjusted for age and sociodemographic factors, was associated with an increased risk of death in men (HR 1.93; 95%CI: 1.05-3.54), with a non-statistically significant association in women (HR 1.85; 95%CI: 0.85-4.04). HIV multimorbidity was also associated with a significantly increased risk of death for both men and women after further adjustment for frailty status.

For peer review only

Table 3. Hazard ratios for risk of death by multimorbidity status

	Any multimorbidity (HR [95% CI])			HIV multimorbidity[a] (HR [95% CI])		
	Total[b] (n=4455)	Women (n=2437)	Men (n=2018)	Total[b] (n=3157)	Women (n=1770)	Men (n=1387)
Model 1	1.56 (1.24-1.97)	1.72 (1.18-2.49)	1.47 (1.10-1.97)	2.08 (1.30-3.35)	1.97 (0.91-4.26)	2.10 (1.15-3.84)
Model 2	1.55 (1.23-1.95)	1.72 (1.18-2.50)	1.46 (1.09-1.95)	1.92 (1.19-3.10)	1.85 (0.85-4.04)	1.93 (1.05-3.54)
Model 3	1.42 (1.13-1.79)	1.55 (1.06-2.26)	1.36 (1.01-1.82)	2.59 (1.57-4.27)	2.75 (1.19-6.37)	2.34 (1.26-4.34)

Model 1-adjusted for age
Model 2-adjusted for age, marital status, education status and socioeconomic status
Model 3-adjusted for age, marital status, education status, socioeconomic status and frailty

[a] adjusted for HIV multimorbidity-time interaction

[b] adjusted for sex and age-sex interaction term

For peer review only

For peer review only - http://bmjopen.bmj.com/site/about/guidelines.xhtml

Results of the odds of death within two years of the data collection are presented in the supplementary files. In summary, in a model fully adjusted for age, socio-demographic factors and frailty, there was a 58% increase in the odds of death within two years in individuals with multimorbidity compared to those without multimorbidity (OR 1.58; 95%CI:1.14-2.19) (Supplementary table 2). The model was able to correctly discriminate between those who did not die within two years and those who did in 76.7% (95%CI: 73.9-79.5%) of the pairs considered, with the addition of frailty resulting in a small but significant improvement in discriminant ability over the model in which multimorbidity was adjusted only for age and sociodemographic factors (Fig. 1).

In fully adjusted models stratified by sex, women with multimorbidity were twice as likely to be dead within two years of the baseline visit as those without multimorbidity (OR 2.05; 95%CI: 1.14-3.69); there was no statistically significant association in men (OR 1.38; 95%CI: 0.92-2.05). The model was able to correctly discriminate between those women who did not die within two years and those who did in 79.2% (95% CI: 74.9-83.6%) of pairs, while in men it correctly discriminated in 72.5% (95%CI: 68.6-76.4%) of cases (Fig. 1). The addition of frailty resulted in a marginal improvement, in women, in discriminant ability over the model in which multimorbidity was adjusted only for age and sociodemographic factors.

For peer review only - http://bmjopen.bmj.com/site/about/guidelines.xhtml

In similarly fully adjusted models, there was a 60% increase in the odds of death within two years in individuals with HIV multimorbidity compared to those with non-HIV multimorbidity (OR 1.60; 95% CI: 1.12-2.30) (Supplementary table 3). Adjustment of HIV multimorbidity for frailty, in addition to age and sociodemographic factors, significantly improved the discriminant ability of the model (Fig 1). In sex-stratified models, there was a 64% increase in the odds of death within two years in men with HIV multimorbidity compared to those with non-HIV multimorbidity (OR 1.64; 95%CI: 1.03-2.61), while in women, the association was not statistically significant. As with any multimorbidity, models that included frailty marginally improved, in women, upon the discriminant ability of models in which HIV multimorbidity was adjusted only for age and sociodemographic factors.

In sensitivity analyses, dyslipidaemia and anaemia, the most frequently missing morbidities, were excluded from the chronic conditions that contributed to the definition of multimorbidity. This resulted in the exclusion of fewer cohort members and there was a final sample size of 4907 individuals, of whom 1719 (35%) had multimorbidity; 637 (37.1%) of these had HIV multimorbidity. Multimorbidity remained significantly associated with a higher risk of death in both women (HR 1.41; 95%CI: 1.07-1.86) and men (HR 1.53; 95%CI: 1.21-1.95) after adjustment for age, sociodemographic factors and frailty. Adjusted HIV multimorbidity, when compared to non-HIV multimorbidity, was associated with a statistically significant marginally higher risk of death in men only (HR 2.13; 95%CI: 1.00-4.57) (Supplementary table 4).

DISCUSSION

For peer review only - http://bmjopen.bmj.com/site/about/guidelines.xhtml

In this older, rural, Black South African cohort, multimorbidity was associated with greater risk of death in both women and men. Men who had HIV as part of their multimorbidity cluster had a higher risk of death than those with non-HIV multimorbidity; the lack of a statistically significant increased risk in women may be because we were underpowered to detect this. The relationship between multimorbidity and death remained robust after adjusting for a range of sociodemographic variables as well as after adjusting for frailty status, itself a strong independent risk factor for mortality. Addition of frailty to models of multimorbidity adjusted for age and other sociodemographic factors slightly increased the ability of these models to discriminate between those who died within two years and those who did not.

Although there is a paucity of literature on multimorbidity and mortality in sub-Saharan Africa, our results are in keeping with similar population-based studies in high-income countries, where several studies report associations between multimorbidity and mortality, and the few studies done in other middle-income countries. One-year mortality rate ratios for individuals ≥ 40 years in a Canadian population ranged between 2 and 6 [32], although in this study, multimorbidity was defined more conservatively as three or more chronic conditions, rather than two. In a Chinese population aged 45 years and older, multimorbidity was associated with a 95% increase in risk of death during a four year follow up [33], while in Brazilian adults ≥60 years, risk for all-cause mortality over 16 years in individuals with two or more chronic diseases ranged between 36% and 105% in women and 58-59% in men [34]. In contrast, an insurance claims-based study in Germans aged 65 years and older identified only a small association between

For peer review only - http://bmjopen.bmj.com/site/about/guidelines.xhtml

multimorbidity and mortality during a 5 year follow up period [35]. This study, however, defined multimorbidity as 2 or more of 46 conditions, some of which were not chronic and would also be expected to have a relatively small effect on mortality, which was reflected in the range of hazard ratios of the individual conditions in the study (0.70-2.29 in women and 0.66-1.95 in men).

This is, to our knowledge, the first study of the relationship between multimorbidity and mortality in sub-Saharan Africa, a region experiencing rapid health transition and with an ageing population and increasing prevalence of multimorbidity. It is also, again to our knowledge, the first study to compare mortality in those with HIV as part of their multimorbidity cluster to those with non-HIV multimorbidity. The study was conducted in a large population-based cohort, using standardised self-reported and objectively assessed morbidities. Our follow up was also robust, with ascertainment of mortality outcome data in over 99% of participants.

Our study was, however, limited by a number of factors. We utilised the definition of multimorbidity proposed by the Academy of Medical Sciences [2], namely of the co-occurrence of two or more chronic conditions. This definition is primarily concerned with the effects of having more than one chronic condition and does not specify which chronic conditions should be considered nor does it give primacy to one type of chronic condition over another. The association between multimorbidity and mortality might therefore differ, depending on the prognoses of the conditions used when defining multimorbidity. Additionally, this definition of multimorbidity does not consider the clinical

For peer review only - http://bmjopen.bmj.com/site/about/guidelines.xhtml

severity or treatment status of the constituent morbidities, other factors which would also be expected to impact mortality. We also considered multimorbidity as a homogeneous concept, and there is some evidence that multimorbidity clusters, defined by groupings of similar diseases [36], are associated with differential mortality risk [37]. Our use of a modified version of the Fried criteria to define frailty, in which we used a threshold body mass index of 18.5 kg/m^2 to define unintentional weight loss, may have increased the prevalence of frailty. Our adoption of the population-specific lowest quintile to identify those with low walk speed, grip strength and physical activity was, however, in keeping with the approach used by Fried and colleagues and our context-specific frailty criteria have, as previously noted, demonstrated construct and predictive validity in this setting, Lastly, we did note a higher proportion of frailty in individuals who were excluded due to missing data which may have diluted the impact of frailty on the relationship between multimorbidity and death. However, in sensitivity analyses in which the most frequently missing individual conditions were omitted from the definition of multimorbidity, the relationship between multimorbidity and mortality persisted.

Our study has implications for both clinical care and health policy in South Africa and other sub-Saharan African countries undergoing health transitions with similar demographics and disease patterns. Clinicians should be aware that the presence of multimorbidity may increase the risk of death in older adults in the short term, even in the absence of impairments in physical function. Aggressive screening and risk factor modification should therefore be employed in individuals with single morbidities who are at high risk of developing a second condition. HIV multimorbidity was associated with

For peer review only - http://bmjopen.bmj.com/site/about/guidelines.xhtml

higher risk of death despite those with HIV multimorbidity being younger and less frail than those without it. This may be due, in part, to only half of those with HIV multimorbidity having an undetectable viral load, with an even lower proportion in those who died, highlighting the need for improved HIV control in the population. The apparent sex differential in the relationship between HIV multimorbidity and mortality merits further discussion. There were fewer deaths among women and our study may therefore have been underpowered to detect a statistically significant association between HIV multimorbidity and mortality in this group. There are also likely other confounders of the relationship between HIV multimorbidity and mortality, such as risk-taking behaviour, which may differ between sexes and were not included in our models. However, when men with HIV had detectable viral loads, these were higher than in women, which may reflect differences in how women and men access HIV care and adhere to treatment and consequently influence mortality [38, 39]. Testing and treatment initiation and adherence campaigns may therefore need to target men more specifically. Our finding that frailty status only modestly enhances the ability of multimorbidity, adjusted for age and sociodemographic factors, to discriminate between those who did and did not die within two years suggests that frailty assessments may be most useful in assessing mortality risk in older adults without multimorbidity.

Our results also have implications for the way in which health systems are structured in South Africa. HIV care frequently serves as the entry point into chronic health care for older adults and adherence to HIV treatment programmes may be associated with improvements in non-communicable disease outcomes [40]. Our findings suggest,

For peer review only - http://bmjopen.bmj.com/site/about/guidelines.xhtml

however, a significant need to broaden the scope of chronic care beyond HIV and ensure that health services are equipped to detect and manage a wide range of chronic conditions in community-dwelling older adults.

CONCLUSION

Our study indicates greater risk of all-cause mortality in older adults with multimorbidity in a sub-Saharan African country. Further research is, however, required to investigate the relationship in this population between mortality and multimorbidity disease clusters as well as the clinical severity of multimorbid conditions. In the interim, models of care which optimise identification and management of multimorbidity, regardless of HIV status, need to be developed [41].

For peer review only

For peer review only - http://bmjopen.bmj.com/site/about/guidelines.xhtml

ACKNOWLEDGEMENTS

We would like to acknowledge the residents of the Agincourt sub-district and Health and socio-Demographic Surveillance System who participated in this study.

MDW acknowledges support from the NIHR Newcastle Biomedical Research Centre.

AUTHOR CONTRIBUTIONS

ANW, LFB, FXGO, CWK, KK, JAS and SMT were involved in design and data collection for HAALSI. SMT, KK, CWK and FXGO established the HDSS and mortality data collection system. ANW, MDW and JID designed this study and ANW performed the statistical analysis. ANW wrote the first draft of the manuscript. ANW, CFP, LFB, AYC, FXGO, CWK, KK, JAS, SMT, MDW and JID critically revised the manuscript and approved the final version.

COMPETING INTERESTS STATEMENT

The authors have no competing interests to report.

FUNDING

This work was supported by the National Institute on Ageing at the National Institutes of Health (grant number 1P01AG041710-01A1); the Department of Science and Innovation, South Africa (no grant number), the University of the Witwatersrand (no grant number), and the Medical Research Council, South Africa (no grant number), and previously the Wellcome Trust, UK (grant numbers 058893/Z/99/A; 069683/Z/02/Z;

For peer review only

For peer review only - http://bmjopen.bmj.com/site/about/guidelines.xhtml

085477/Z/08/Z; 085477/B/08/Z) to the MRC/Wits Rural Public Health and Health Transitions Research Unit and Agincourt Health and socio-Demographic Surveillance System, a node of the South African Population Research Infrastructure Network (SAPRIN); the Fogarty International Centre of the National Institutes of Health (grant number K43TW010698) to ANW; the Australian Research Council Discovery Early Career Award (DE210100087) and the Australian National University Futures Scheme (no grant number) to CFP.

This paper describes the views of the authors and does not necessarily represent the official views of the National Institutes of Health (USA).

The funders had no role in study design, data collection, analysis and interpretation, report writing or the decision to submit this article for publication.

DATA SHARING STATEMENT

The HAALSI baseline data are publicly available at the Harvard Centre for Population and Development Studies (HCPDS) programme website [www.haalsi.org]. Data are also accessible through the Inter-university Consortium for Political and Social Research (ICPSR) at the University of Michigan [www.icpsr.umich.edu] and the INDEPTH Data Repository [http://www.indepth-ishare.org/index.php/catalog/113]. Mortality data are available upon request.

For peer review only - http://bmjopen.bmj.com/site/about/guidelines.xhtml

REFERENCES

1. Barnett K, Mercer SW, Norbury M, et al. Epidemiology of multimorbidity and implications for health care, research, and medical education: a cross-sectional study. *Lancet.* 2012;380:37-43. doi: 10.1016/S0140-6736(12)60240-2.

2. Multimorbidity: a priority for global health research. London: Academy of Medical Sciences;2018.

3. Chang AY, Gómez-Olivé FX, Payne C, et al. Chronic multimorbidity among older adults in rural South Africa. *BMJ Glob Health.* 2019;4:e001386. doi: 10.1136/bmjgh-2018-001386.

4. Nimako BA, Baiden F, Sackey SO, et al. Multimorbidity of chronic diseases among adult patients presenting to an inner-city clinic in Ghana. *Global Health.* 2013;9:61. doi: 10.1186/1744-8603-9-61.

5. Hien H, Berthe A, Drabo MK, et al. Prevalence and patterns of multimorbidity among the elderly in Burkina Faso: cross-sectional study. *Trop Med Int Health.* 2014;19:1328-1333. doi: 10.1111/tmi.12377.

6. Gouda HN, Charlson F, Sorsdahl K, et al. Burden of non-communicable diseases in sub-Saharan Africa, 1990-2017: results from the Global Burden of Disease Study 2017. *Lancet Glob Health.* 2019;7:e1375-e1387. doi: 10.1016/S2214-109X(19)30374-2.

7. Garin N, Koyanagi A, Chatterji S, et al. Global multimorbidity patterns: a cross-sectional, population-based, multi-country study. *J Gerontol A Biol Sci Med Sci.* 2016;71:205-214. doi: 10.1093/gerona/glv128.

For peer review only

8. Odland ML, Payne C, Witham MD, et al. Epidemiology of multimorbidity in conditions of extreme poverty: a population-based study of older adults in rural Burkina Faso. *BMJ Glob Health.* 2020;5:e002096. doi: 10.1136/bmjgh-2019-002096.

9. Magodoro IM, Esterhuizen TM, Chivese T. A cross-sectional, facility based study of comorbid non-communicable diseases among adults living with HIV infection in Zimbabwe. *BMC Res Notes.* 2016;9:379. doi: 10.1186/s13104-016-2187-z.

10. Kansiime S, Mwesigire D, Mugerwa H. Prevalence of non-communicable diseases among HIV positive patients on antiretroviral therapy at Joint Clinical Research Centre, Lubowa, Uganda. *PLoS One.* 2019;14:e0221022. doi: 10.1371/journal.pone.0221022.

11. Oni T, Youngblood E, Boulle A, et al. Patterns of HIV, TB, and non-communicable disease multi-morbidity in peri-urban South Africa- a cross sectional study. *BMC Infect Dis.* 2015;15:20. doi: 10.1186/s12879-015-0750-1.

12. Nunes BP, Flores TR, Mielke GI, et al. Multimorbidity and mortality in older adults: a systematic review and meta-analysis. *Arch Gerontol Geriatr.* 2016;67:130-138. doi: 10.1016/j.archger.2016.07.008.

13. Manne-Goehler J, Montana L, Gomez-Olive FX, et al. The ART advantage: health care utilization for diabetes and hypertension in rural South Africa. *J Acquir Immune Defic Syndr.* 2017;75:561-567. doi: 10.1097/QAI.0000000000001445.

14. Clegg A, Young J, Iliffe S, et al. Frailty in elderly people. *Lancet.* 2013;381:752-762. doi: 10.1016/S0140-6736(12)62167-9.

For peer review only

For peer review only - http://bmjopen.bmj.com/site/about/guidelines.xhtml

15. Majid Z, Welch C, Davies J, et al. Global frailty: the role of ethnicity, migration and socioeconomic factors. *Maturitas.* 2020;139:33-41. doi: 10.1016/j.maturitas.2020.05.010.

16. Payne CF, Wade A, Kabudula CW, et al. Prevalence and correlates of frailty in an older rural African population: findings from the HAALSI cohort study. *BMC Geriatr.* 2017;17:293. doi: 10.1186/s12877-017-0694-y.

17. Payne CF, Gomez-Olive FX, Kahn K, et al. Physical function in an ageing population in rural South Africa: findings from HAALSI and cross-national comparisons with HRS sister studies. *J Gerontol B Psychol Sci Soc Sci.* 2017;72:665-679. doi: 10.1093/geronb/gbx030.

18. Gomez-Olive FX, Montana L, Wagner RG, et al. Cohort profile: Health and Ageing in Africa: a Longitudinal Study of an INDEPTH Community in South Africa (HAALSI). Int J Epidemiol. 2018;47:689-690j. doi: 10.1093/ije/dyx247.

19. Kahn K, Collinson MA, Gomez-Olive FX, et al. Profile: Agincourt health and socio-demographic surveillance system. *Int J Epidemiol.* 2012;41:988-1001. doi: 10.1093/ije/dys115.

20. Vyas S, Kumaranayake L. Constructing socio-economic status indices: how to use principal components analysis. *Health Policy Plan.* 2006;21:459-468. doi: 10.1093/heapol/czl029.

21. Chobanian AV, Bakris GL, Black HR, et al. Seventh report of the Joint National Committee on prevention, detection, evaluation, and treatment of high blood pressure. *Hypertension.* 2003;42:1206-1252. doi: 10.1161/01.HYP.0000107251.49515.c2.

For peer review only - http://bmjopen.bmj.com/site/about/guidelines.xhtml

22. American Diabetes Association. Classification and diagnosis of diabetes: standards of medical care in diabetes-2020. *Diabetes Care.* 2020;43(Suppl 1):S14-S31. doi: 10.2337/dc20-S002.

23. Shisana O, Labadarios D, Rehle T, et al. South African National Health and Nutrition Examination Survey (SANHANES-1). Cape Town: HSRC Press; 2013.

24. Rose G, McCartney P, Reid DD. Self-administration of a questionnaire on chest pain and intermittent claudication. *Br J Prev Soc Med.* 1977;31:42-48. doi: 10.1136/jech.31.1.42.

25. Ehrlich RI, White N, Norman R, et al. Predictors of chronic bronchitis in South African adults. *Int J Tuberc Lung Dis.* 2004;8:369-376.

26. Radloff LS. The CES-D scale:a self-report depression scale for research in the general population. *Appl Psychol Meas.* 1977;1:385-401. doi: 10.1177/014662167700100306.

27. Breslau N, Peterson EL, Kessler RC, et al. Short screening scale for DSM-IV posttraumatic stress disorder. *Am J Psychiatry.* 1999;156:908-911. doi: 10.1176/ajp.156.6.908.

28. Ewing JA. Detecting alcoholism. The CAGE questionnaire. *JAMA.* 1984;252:1905-1907. doi: 10.1001/jama.252.14.1905.

29. Fried LP, Tangen CM, Walston J, et al. Frailty in older adults: evidence for a phenotype. *J Gerontol A Biol Sci Med Sci.* 2001;56:M146-156.

30. World Health Organisation. Global Physical Activity Questionnaire analysis guide. https://www.who.int/ncds/surveillance/steps/GPAQ/en/. Accessed October 30, 2020.

For peer review only

For peer review only - http://bmjopen.bmj.com/site/about/guidelines.xhtml

31. Gómez-Olivé FX, Thorogood M, Bocquier P, et al. Social conditions and disability related to the mortality of older people in rural South Africa. *Int J Epidemiol.* 2014;43:1531-1541. doi: 10.1093/ije/dyu093.

32. Ryan BL, Allen B, Zwarenstein M, et al. Multimorbidity and mortality in Ontario, Canada: a population-based retrospective cohort study. *J Comorb.* 2020;10:2235042X20950598. doi: 10.1177/2235042X20950598.

33. Zhang Y, Zhou L, Liu S, et al. Prevalence, correlates and outcomes of multimorbidity among the middle-aged and elderly: findings from the China Health and Retirement Longitudinal Study. *Arch Gerontol Geriatr.* 2020;90:104135. doi: 10.1016/j.archger.2020.104135.

34. Roman Lay AA, Ferreira do Nascimento C, Caba Burgos F, et al. Gender differences between multimorbidity and all-cause mortality among older adults. *Curr Gerontol Geriatr Res.* 2020;2020:7816785. doi: 10.1155/2020/7816785.

35. Schafer I, Kaduszkiewicz H, Nguyen TS, et al. Multimorbidity patterns and 5-year overall mortality: results from a claims data-based observational study. *J Comorb.* 2018;8:2235042X18816588. doi: 10.1177/2235042X18816588.

36. Chang AY, Gomez-Olive FX, Manne-Goehler J, et al. Multimorbidity and care for hypertension, diabetes and HIV among older adults in rural South Africa. *Bull World Health Organ.* 2019;97:10-23. doi: 10.2471/BLT.18.217000.

37. Vetrano DL, Roso-Llorach A, Fernandez S, et al. Twelve-year clinical trajectories of multimorbidity in a population of older adults. *Nat Commun.* 2020;11:3223. doi: 10.1038/s41467-020-16780-x.

For peer review only - http://bmjopen.bmj.com/site/about/guidelines.xhtml

38. Osler M, Cornell M, Ford N, et al. Population-wide differentials in HIV service access and outcomes in the Western Cape for men as compared to women, South Africa: 2008 to 2018: a cohort analysis. *J Int AIDS Soc.* 2020;23 Suppl 2:e25530. doi: 10.1002/jia2.25530.

39. Madut DB, Park LP, Yao J, et al. Predictors of mortality in treatment experienced HIV-infected patients in northern Tanzania. *PLoS One.* 2020;15:e0240293. doi: 10.1371/journal.pone.0240293.

40. Manne-Goehler J, Siedner MJ, Montana L, et al. Hypertension and diabetes control along the HIV care cascade in rural South Africa. *J Int AIDS Soc.* 2019;22:e25213. doi: 10.1002/jia2.25213.

41. Ameh S, Gomez-Olive FX, Kahn K, et al. Multilevel predictors of controlled CD4 count and blood pressure in an integrated chronic disease management model in rural South Africa: a panel study. *BMJ Open.* 2020;10:e037580. doi: 10.1136/bmjopen-2020-037580.

For peer review only

Figure 1. Receiver operating curves for multimorbidity and death within two years

Model 1- any multimorbidity/HIV multimorbidity and age; model 2-any multimorbidity/HIV multimorbidity, marital status, educational attainment and socioeconomic status; model 3-any multimorbidity/HIV multimorbidity, marital status, educational attainment, socioeconomic status and frailty status. Models constructed for total sample were additionally adjusted for sex. AUC: area under the curve

For peer review only

For peer review only - http://bmjopen.bmj.com/site/about/guidelines.xhtml

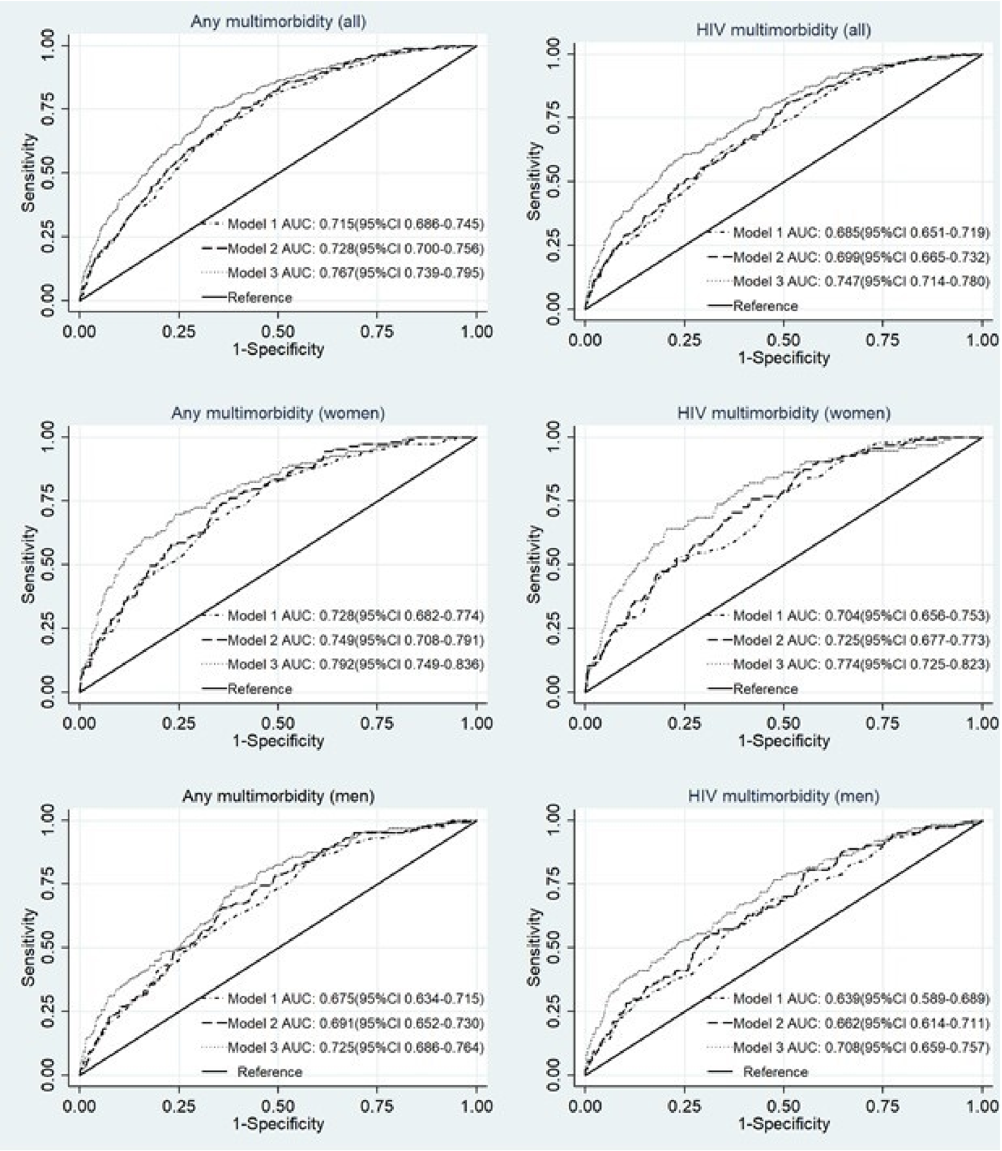

Figure 1. Receiver operating curves for multimorbidity and death within two years
Model 1- any multimorbidity/HIV multimorbidity and age; model 2-any multimorbidity/HIV multimorbidity, marital status, educational attainment and socioeconomic status; model 3-any multimorbidity/HIV multimorbidity, marital status, educational attainment, socioeconomic status and frailty status. Models constructed for total sample were additionally adjusted for sex. AUC: area under the curve

163x189mm (96 x 96 DPI)

Supplementary table 1. Frequency of individual morbidities in individuals with multimorbidity, overall and by sex, age group and HIV status

	Multimorbidity	Sex		Age group (years)					Non-HIV multimorbidity	HIV multimorbidity
		Women	Men	40-49	50-59	60-69	70-79	80+		
	n=3157	n=1770	n=1387	n=507	n=885	n=855	n=555	n=355	n=2242	n=915
Hypertension (%)	2395 (75.9)	1393 (78.7)	1002 (72.2)	292 (57.6)	652 (73.7)	678 (79.3)	467 (84.1)	306 (86.2)	1909 (85.2)	486 (53.1)
Diabetes (%)	534 (16.9)	309 (17.5)	225 (16.2)	46 (9.1)	135 (15.3)	165 (19.3)	123 (22.2)	65 (18.3)	455 (20.3)	79 (8.6)
Dyslipidaemia (%)	1686 (58.1)	919 (56.1)	767 (60.6)	287 (61.6)	480 (59.3)	479 (60.2)	276 (53.1)	164 (52.7)	1280 (62.2)	406 (48.1)
Anaemia (%)	1655 (55.2)	939 (56.2)	716 (53.9)	274 (56.3)	438 (52.1)	445 (54.9)	302 (58.4)	196 (57.3)	1126 (53.3)	529 (59.7)
HIV (%)	915 (29.0)	509 (28.8)	406 (29.3)	256 (50.5)	346 (39.1)	222 (26.0)	79 (14.2)	12 (3.4)		
Angina (%)	428 (13.6)	281 (15.9)	147 (10.6)	47 (9.3)	119 (13.5)	127 (14.9)	74 (13.3)	61 (17.2)	350 (15.6)	78 (8.5)
Chronic bronchitis (%)	27 (0.9)	12 (0.7)	15 (1.1)	5 (1.0)	9 (1.0)	5 (0.6)	4 (0.7)	4 (1.1)	18 (0.8)	9 (1.0)
Depression (%)	752 (24.2)	450 (25.8)	302 (22.1)	78 (15.5)	159 (18.2)	213 (25.2)	156 (28.8)	146 (42.3)	613 (27.8)	139 (15.3)
Post-traumatic stress disorder (%)	222 (7.1)	141 (8.1)	81 (5.9)	31 (6.2)	65 (7.4)	58 (6.9)	39 (7.2)	29 (8.4)	173 (7.9)	49 (5.4)
Alcohol dependence (%)	56 (1.8)	10 (0.6)	46 (3.3)	9 (1.8)	16 (1.8)	24 (2.8)	5 (0.9)	2 (0.6)	42 (1.9)	14 (1.5)

254 individuals had missing data on dyslipidaemia

159 individuals had missing data on anaemia

2 individuals had missing data on chronic bronchitis

49 individuals had missing data on depression

48 individuals had missing data on post-traumatic stress disorder

1 individual had missing data on alcohol dependence

For peer review only

For peer review only - http://bmjopen.bmj.com/site/about/guidelines.xhtml

Supplementary table 2. Odds ratios for death within two years for any multimorbidity vs no multimorbidity

	Total (n=4452)[a]			Women (n=2435)			Men (n=2017)		
	Model 1	Model 2	Model 3	Model 1	Model 2	Model 3	Model 1	Model 2	Model 3
Any multimorbidity	1.77 (1.29-2.44)	1.75 (1.27-2.41)	1.58 (1.14-2.19)	2.40 (1.35-4.25)	2.34 (1.32-4.16)	2.05 (1.14-3.69)	1.51 (1.02-2.23)	1.51 (1.02-2.24)	1.38 (0.92-2.05)
Male sex	1.88 (1.46-2.43)	2.33 (1.74-3.11)	2.33 (1.73-3.14)						
Age	1.05 (1.04-1.06)	1.05 (1.04-1.06)	1.03 (1.02-1.05)	1.06 (1.04-1.07)	1.05 (1.03-1.07)	1.03 (1.01-1.05)	1.05 (1.03-1.06)	1.05 (1.03-1.06)	1.04 (1.02-1.05)
Marital status									
Never married		Reference	Reference		Reference	Reference		Reference	Reference
Previously married		1.06 (0.51-2.18)	1.28 (0.61-2.68)		1.50 (0.35-6.40)	1.62 (0.37-7.07)		0.87 (0.37-2.07)	1.09 (0.45-2.61)
Currently married		0.67 (0.33-1.38)	0.88 (0.42-1.83)		0.67 (0.15-3.01)	0.79 (0.17-3.62)		0.67 (0.29-1.55)	0.88 (0.38-2.05)
Educational attainment									
No formal education		Reference	Reference		Reference	Reference		Reference	Reference
Some primary education		0.92 (0.69-1.24)	1.01 (0.74-1.37)		0.86 (0.53-1.40)	1.01 (0.62-1.66)		0.97 (0.66-1.42)	1.03 (0.70-1.52)
Some secondary education		1.02 (0.62-1.67)	1.13 (0.68-1.88)		1.07 (0.42-2.73)	1.27 (0.49-3.28)		1.03 (0.57-1.86)	1.12 (0.61-2.05)
Secondary or more		0.31 (0.11-0.87)	0.28 (0.10-0.82)		0.26 (0.03-1.96)	0.24 (0.03-1.86)		0.35 (0.10-1.18)	0.33 (0.10-1.13)
Wealth index quintile									
First		Reference	Reference		Reference	Reference		Reference	Reference
Second		1.13 (0.77-1.66)	1.06 (0.72-1.57)		1.17 (0.65-2.13)	1.14 (0.62-2.10)		1.10 (0.67-1.83)	1.03 (0.62-1.72)
Third		1.13 (0.76-1.68)	1.05 (0.70-1.57)		1.11 (0.60-2.05)	0.95 (0.50-1.79)		1.13 (0.68-1.89)	1.10 (0.66-1.86)
Fourth		0.92 (0.61-1.40)	0.94 (0.62-1.44)		0.84 (0.43-1.66)	0.79 (0.40-1.58)		0.98 (0.57-1.66)	1.05 (0.61-1.80)
Fifth		1.18 (0.77-1.80)	1.25 (0.81-1.93)		1.65 (0.86-3.15)	1.68 (0.86-3.26)		0.94 (0.53-1.65)	1.01 (0.57-1.79)
Frailty status									
Non-frail			Reference			Reference			Reference
Pre-frail			1.38 (1.01-1.91)			1.28 (0.76-2.18)			1.49 (1.00-2.24)
Frail			4.72 (3.32-6.71)			5.54 (3.14-9.77)			4.22 (2.67-6.68)

Estimates are given as odds ratios and 95% confidence intervals
Model 1-adjusted for age and sex (in models constructed for total sample)
Model 2-adjusted for age, sex (in models constructed for total sample), marital status, educational attainment and socioeconomic status
Model 3-adjusted for age, sex (in models constructed for total sample), marital status, educational attainment, socioeconomic status and frailty
[a]3 individuals lost to follow up were excluded from the analysis

For peer review only

For peer review only - http://bmjopen.bmj.com/site/about/guidelines.xhtml

Supplementary table 3. Odds ratios for death within two years for HIV multimorbidity vs non-HIV multimorbidity

	Total (n=3155)[a]			Women (n=1763)			Men (n=1385)		
	Model 1	Model 2	Model 3	Model 1	Model 2	Model 3	Model 1	Model 2	Model 3
HIV multimorbidity	1.53 (1.09-2.15)	1.41 (0.99-2.00)	1.60 (1.12-2.30)	1.51 (0.87-2.60)	1.36 (0.78-2.39)	1.55 (0.87-2.77)	1.55 (1.00-2.40)	1.48 (0.94-2.34)	1.64 (1.03-2.61)
Male sex	1.71 (1.29-2.27)	1.97 (1.43-2.72)	1.94 (1.39-2.70)						
Age	1.05 (1.04-1.07)	1.05 (1.03-1.06)	1.04 (1.02-1.05)	1.06 (1.04-1.08)	1.05 (1.03-1.08)	1.04 (1.02-1.06)	1.04 (1.03-1.06)	1.04 (1.02-1.06)	1.03 (1.01-1.05)
Marital status									
Never married		Reference	Reference		Reference	Reference		Reference	Reference
Previously married		0.76 (0.36-1.61)	0.91 (0.42-1.96)		1.16 (0.27-5.08)	1.29 (0.29-5.75)		0.62 (0.25-1.53)	0.77 (0.30-1.97)
Currently married		0.57 (0.27-1.19)	0.76 (0.36-1.61)		0.63 (0.14-2.90)	0.79 (0.17-3.74)		0.58 (0.24-1.37)	0.77 (0.32-1.87)
Educational attainment									
No formal education		Reference	Reference		Reference	Reference		Reference	Reference
Some primary education		0.74 (0.53-1.05)	0.83 (0.58-1.18)		0.88 (0.52-1.49)	1.04 (0.61-1.79)		0.67 (0.42-1.05)	0.71 (0.45-1.14)
Some secondary education		1.22 (0.73-2.04)	1.42 (0.84-2.42)		1.32 (0.51-3.42)	1.61 (0.61-4.23)		1.19 (0.64-2.22)	1.35 (0.71-2.57)
Secondary or more		0.35 (0.12-1.00)	0.33 (0.11-0.97)		0.32 (0.04-2.47)	0.32 (0.04-2.52)		0.36 (0.10-1.26)	0.34 (0.10-1.21)
Wealth index quintile									
First		Reference	Reference		Reference	Reference		Reference	Reference
Second		1.14 (0.74-1.75)	1.04 (0.67-1.62)		1.18 (0.63-2.23)	1.16 (0.61-2.22)		1.07 (0.60-1.93)	0.94 (0.51-1.71)
Third		1.11 (0.71-1.73)	0.97 (0.62-1.53)		0.99 (0.51-1.93)	0.84 (0.42-1.67)		1.19 (0.65-2.16)	1.07 (0.58-1.98)
Fourth		0.96 (0.60-1.52)	0.96 (0.60-1.54)		0.81 (0.39-1.67)	0.77 (0.37-1.60)		1.07 (0.58-1.97)	1.12 (0.60-2.09)
Fifth		1.24 (0.77-2.00)	1.31 (0.80-2.13)		1.58 (0.78-3.17)	1.60 (0.78-3.27)		1.02 (0.53-1.97)	1.11 (0.57-2.16)
Frailty status									
Non-frail			Reference			Reference			Reference
Pre-frail			1.41 (0.98-2.02)			1.42 (0.80-2.50)			1.43 (0.89-2.28)
Frail			5.20 (3.50-7.72)			5.42 (2.90-10.16)			5.06 (3.02-8.50)

Estimates are given as odds ratios and 95% confidence intervals

Model 1-adjusted for age and sex (in models constructed for total sample)

Model 2-adjusted for age, sex (in models constructed for total sample), marital status, educational attainment and socioeconomic status

Model 3-adjusted for age, sex (in models constructed for total sample), marital status, educational attainment, socioeconomic status and frailty

[a]2 individuals lost to follow up were excluded from the analysis

For peer review only - http://bmjopen.bmj.com/site/about/guidelines.xhtml

Supplementary table 4. Hazard ratios for time to death by multimorbidity status- dyslipidaemia and anaemia excluded from multimorbidity definition

	Any multimorbidity (HR [95% CI])			HIV multimorbidity[a] (HR [95% CI])		
	Total[b] (n=4907)	Women (n=2637)	Men (n=2270)	Total[b] (n=1719)	Women (n=1019)	Men (n=700)
Model 1	1.58 (1.32-1.89)	1.50 (1.14-1.97)	1.64 (1.29-2.07)	1.32 (0.73-2.37)	0.89 (0.31-2.52)	1.51 (0.73-3.10)
Model 2	1.57 (1.31-1.88)	1.50 (1.14-1.98)	1.63 (1.29-2.07)	1.23 (0.68-2.22)	0.83 (0.29-2.36)	1.41 (0.68-2.94)
Model 3	1.47 (1.23-1.77)	1.41 (1.07-1.86)	1.53 (1.21-1.95)	1.90 (1.02-3.57)	1.13 (0.36-3.49)	2.13 (1.00-4.57)

Model 1-adjusted for age

Model 2-adjusted for age, marital status, education status and socioeconomic status

Model 3-adjusted for age, marital status, education status, socioeconomic status and frailty

[a] adjusted for HIV multimorbidity-time interaction

[b] adjusted for sex and age-sex interaction term

For peer review only - http://bmjopen.bmj.com/site/about/guidelines.xhtml

STROBE 2007 (v4) Statement—Checklist of items that should be included in reports of *cohort studies*

**Section/Topic**	**Item #**	**Recommendation**	**Reported on page #**
**Title and abstract**	1	(*a*) Indicate the study's design with a commonly used term in the title or the abstract	1
		(*b*) Provide in the abstract an informative and balanced summary of what was done and what was found	3-4
**Introduction**			
Background/rationale	2	Explain the scientific background and rationale for the investigation being reported	6-7
Objectives	3	State specific objectives, including any prespecified hypotheses	7
**Methods**			
Study design	4	Present key elements of study design early in the paper	7-8
Setting	5	Describe the setting, locations, and relevant dates, including periods of recruitment, exposure, follow-up, and data collection	7-11
Participants	6	(*a*) Give the eligibility criteria, and the sources and methods of selection of participants. Describe methods of follow-up	7-8
		(*b*) For matched studies, give matching criteria and number of exposed and unexposed	n/a
Variables	7	Clearly define all outcomes, exposures, predictors, potential confounders, and effect modifiers. Give diagnostic criteria, if applicable	8-11
Data sources/ measurement	8*	For each variable of interest, give sources of data and details of methods of assessment (measurement). Describe comparability of assessment methods if there is more than one group	8-11
Bias	9	Describe any efforts to address potential sources of bias	13
Study size	10	Explain how the study size was arrived at	8,14
Quantitative variables	11	Explain how quantitative variables were handled in the analyses. If applicable, describe which groupings were chosen and why	11-12
Statistical methods	12	(*a*) Describe all statistical methods, including those used to control for confounding	11-13
		(*b*) Describe any methods used to examine subgroups and interactions	11-12
		(*c*) Explain how missing data were addressed	14
		(*d*) If applicable, explain how loss to follow-up was addressed	14
		(*e*) Describe any sensitivity analyses	13
**Results**			

For peer review only

Participants	13*	(a) Report numbers of individuals at each stage of study—eg numbers potentially eligible, examined for eligibility, confirmed eligible, included in the study, completing follow-up, and analysed	14
		(b) Give reasons for non-participation at each stage	14
		(c) Consider use of a flow diagram	
Descriptive data	14*	(a) Give characteristics of study participants (eg demographic, clinical, social) and information on exposures and potential confounders	14-16
		(b) Indicate number of participants with missing data for each variable of interest	14
		(c) Summarise follow-up time (eg, average and total amount)	15
Outcome data	15*	Report numbers of outcome events or summary measures over time	15, 17
Main results	16	(*a*) Give unadjusted estimates and, if applicable, confounder-adjusted estimates and their precision (eg, 95% confidence interval). Make clear which confounders were adjusted for and why they were included	18-21
		(*b*) Report category boundaries when continuous variables were categorized	n/a
		(*c*) If relevant, consider translating estimates of relative risk into absolute risk for a meaningful time period	n/a
Other analyses	17	Report other analyses done—eg analyses of subgroups and interactions, and sensitivity analyses	21
**Discussion**			
Key results	18	Summarise key results with reference to study objectives	22
**Limitations**			
Interpretation	20	Give a cautious overall interpretation of results considering objectives, limitations, multiplicity of analyses, results from similar studies, and other relevant evidence	22-25
Generalisability	21	Discuss the generalisability (external validity) of the study results	23-24
**Other information**			
Funding	22	Give the source of funding and the role of the funders for the present study and, if applicable, for the original study on which the present article is based	27-28

*Give information separately for cases and controls in case-control studies and, if applicable, for exposed and unexposed groups in cohort and cross-sectional studies.

Note: An Explanation and Elaboration article discusses each checklist item and gives methodological background and published examples of transparent reporting. The STROBE checklist is best used in conjunction with this article (freely available on the Web sites of PLoS Medicine at http://www.plosmedicine.org/, Annals of Internal Medicine at http://www.annals.org/, and Epidemiology at http://www.epidem.com/). Information on the STROBE Initiative is available at www.strobe-statement.org.

For peer review only
